# Induced seismicity response of hydraulic fracturing: results of a multidisciplinary monitoring at the Wysin site, Poland

**DOI:** 10.1038/s41598-018-26970-9

**Published:** 2018-06-05

**Authors:** J. A. López-Comino, S. Cesca, J. Jarosławski, N. Montcoudiol, S. Heimann, T. Dahm, S. Lasocki, A. Gunning, P. Capuano, W. L. Ellsworth

**Affiliations:** 1 GFZ German Research Centre for Geosciences, Telegrafenberg, D-14473 Potsdam, Germany; 2 Institute of Geophysics, Polish Academy of Sciences, ul. Ksiecia Janusza 64, PL-01-452 Warsaw, Poland; 3 School of Engineering, University of Glasgow, G12 8QQ Glasgow, United Kingdom; 4 RSKW Ltd, Stirling, United Kingdom; 5 Dipartimento di Fisica, Università degli Studiy di Salerno, Fisciano, Italy; 6 Department of Geophysics, Stanford University, Stanford, USA

## Abstract

Shale oil and gas exploitation by hydraulic fracturing experienced a strong development worldwide over the last years, accompanied by a substantial increase of related induced seismicity, either consequence of fracturing or wastewater injection. In Europe, unconventional hydrocarbon resources remain underdeveloped and their exploitation controversial. In UK, fracturing operations were stopped after the M_w_ 2.3 Blackpool induced earthquake; in Poland, operations were halted in 2017 due to adverse oil market conditions. One of the last operated well at Wysin, Poland, was monitored independently in the framework of the EU project SHEER, through a multidisciplinary system including seismic, water and air quality monitoring. The hybrid seismic network combines surface mini-arrays, broadband and shallow borehole sensors. This paper summarizes the outcomes of the seismological analysis of these data. Shallow artificial seismic noise sources were detected and located at the wellhead active during the fracturing stages. Local microseismicity was also detected, located and characterised, culminating in two events of M_w_ 1.0 and 0.5, occurring days after the stimulation in the vicinity of the operational well, but at very shallow depths. A sharp methane peak was detected ~19 hours after the M_w_ 0.5 event. No correlation was observed between injected volumes, seismicity and groundwater parameters.

## Introduction

Hydraulic fracturing (HF), or fracking, is a technique designed to recover gas and oil from so-called unconventional reservoirs, which correspond to tight sands, coal beds or shale formations. The exploitation performance is improved applying HF techniques, where high-pressure fluid, generally a mixture of water, sand and chemical proppants, is injected into the boreholes in order to enhance the permeability of the formation in contact with the well bore. The fracturing process starts when the stress on the hole wall in the direction of the maximum *in situ* stress exceeds the tensile strength of rock^1–3^. The permeability into the surrounding rocks is increased by the creation of new hydraulic fractures and reactivation of well-oriented pre-existing faults and fractures. Small grains of proppants are pumped into the newly opened fractures to hold them open, allowing gas and oil to flow out to the wellhead.

Over the last decades, HF has generated a large amount of controversy, since the deployment of high-volume HF potentially entails some risk to the environment. In Europe, the potential application of this technology has led to worries regarding the alleged magnitude of the environmental impact, and expectations about production of hydrocarbons. The first UK exploration for shale gas using HF was suspended at Blackpool after a M_w_ 2.3 induced earthquake, on April 1^st^, 2011^4^, drawing significantly the public attention to the problem of HF induced seismicity. In Poland, early HF operations were halted in 2017 due to adverse oil market conditions and disappointing results from the exploration phase due to the geology. The potential environmental impact of HF operations has resulted in a temporary HF moratorium in most European countries. The main concerns to HF are the potential contaminate of groundwater at the fracking site due to the injection of proppants, air pollution resulting by HF operations, and induced seismicity. In this paper, we focus on the HF consequences mostly in terms of induced microseismicity and we discuss the results of the seismological monitoring and analysis at the Wysin site, Poland.

Induced seismicity generally refers to earthquakes related to industrial processes and anthropogenic operations^5–7^. Among the human activities which can induce and trigger seismicity, such as water reservoir impoundment, groundwater extraction, mining, wastewater disposal, oil and gas extraction, natural gas storage and geothermal field stimulation, HF plays an important role. The induced seismic hazard of HF concerns direct and indirect effects of shale gas exploitation. HF can directly stimulate seismicity through injection of pressurized fluid, by the formation and growth of tensile fractures and by affecting the pore pressure and stress conditions in underground formations, and the consequent (re)activation of local faults.

The most numerous and recent cases of induced seismicity which have been directly associated to HF, with a highly correlation in time and space with fracturing wells, were located in the Western Canada Sedimentary Basin (WCSB)^8^. Between 2009 and 2011, events ranging in local magnitude (M_L_) between 2.2 and 3.8 were observed in northeast British Columbia^9^. Larger events were recorded in 2014: a M_w_ 4.0 and a M_w_ 4.2 near Fort St. John, British Columbia, and a M_w_ 3.9 near Rocky Mountain House, Alberta^10^. However, the largest event ever related to HF operations occurred on August 17^th^, 2015, near Fort St. John, British Columbia, with a M_w_ 4.6^11^; although we note that magnitudes up to M_w_ 4.7 have been reported in the Sichuan Basin (China) involving injection-induced fault reactivation^12^. Other relevant cases have also been reported in the United States of America. In south-central Oklahoma, earthquakes ranging in local magnitude from M_L_ 0.6 to 2.9 were identified in January 2011, which were likely triggered by HF operations^13^. A small earthquake sequence of 10 events (up to a maximum magnitude M_w_ 2.2) located at Harrison County (Ohio) in October 2013 were linked to HF operations at the nearby Ryser wells^14^. Between 4 and 12 March 2014, a serie of 77 earthquakes with M_L_ ~1.0 up to 3.0 in Poland Township (Ohio) were related to HF operations, causing a shutdown of HF at a nearby well on 10 March, immediately after the largest M_L_ 3.0 seismic event^15^. Recent works studied the seismicity associated with the fracking of 53 wells and initiation of wastewater injection over a 3-month period in 2010 in the Guy-Greenbrier, Arkansas area^16^. Their results showed that only about half of the stimulated wells induced seismicity at a detection threshold below M_L_ 0. At several of the wells that induced earthquakes seismicity persisted for weeks after the completion of hydraulic fracturing operations. Few produces earthquakes as large as M_L_ 2.0, with a maximum observed event of M_-_ 2.9. Clearly, there is substantial variability in the seismic response to fracking, both regionally and within a single field.

While few cases have been observed in Europe, in recent years some initiatives have emerged in order to mitigate and characterize the seismic activity related with the fluid injection processes. The most significant case of European HF induced seismicity struck near Blackpool, UK, on April 1^st^, 2011, corresponding to the first felt shale-gas related HF induced earthquake in Europe including 52 seismic events with local magnitudes between M_L_ −2 and 2.3^4^. Furthermore, a seismic analysis of small-scale HF experiments has been conducted in underground mines^17–19^, at the Äspö Hard Rock Laboratory (Sweden) and the Deep Underground Geothermal Laboratory (DUG-Lab) at Grimsel (Switzerland), with the purpose of characterize the growth of tensile fracture and magnitude distributions in controlled HF experiments. Following the Äspö experiment, the fracture growth has been mapped through the detection and location of acoustic emission events with M_w_ < −3.0^20,21^. In conclusion the amount of induced seismicity following HF operations varies substantially both within and across sites in terms of number of events and maximal magnitude. Generally, these operations induce weak microseismic events with reported moment magnitudes below 0^22–25^, which are often challenging to detect and locate with surface installations alone. However, in some cases HF has been considered responsible for triggering moderate earthquakes up to M_w_ > 4, which have caused important material damages and causalities^11^.

**Figure 1:**
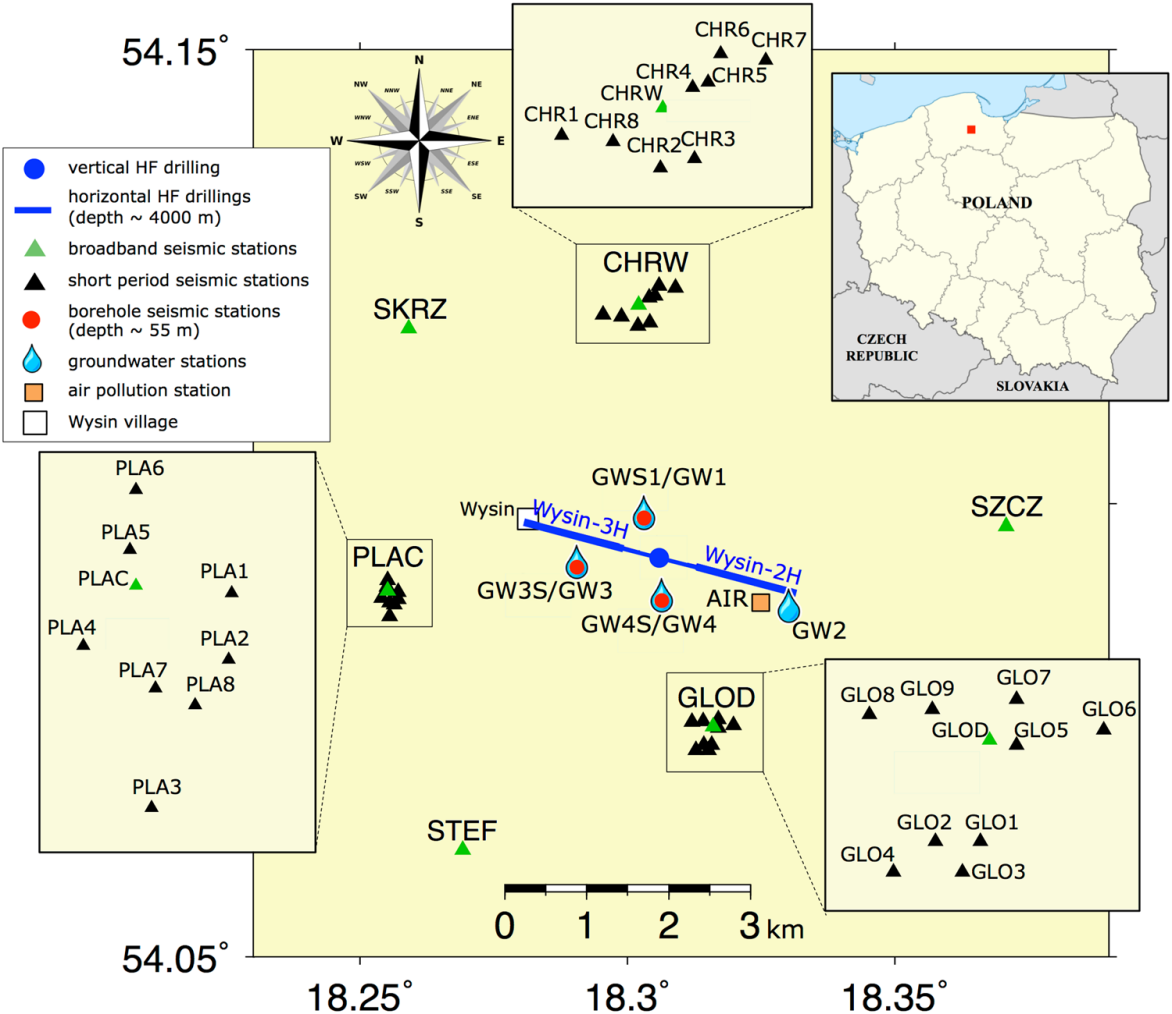
Map of seismic, air and groundwater monitoring at the Wysin site (Poland). The seismic monitoring includes broad-band stations (green triangles), small-scale arrays (inset boxes) composed by 8–9 short-period stations each (black triangles), and borehole stations (red circles). The air pollution station (orange square) is located at Stary Wiec village. Groundwater borehole monitoring stations are denoted by water drop symbols; some of them are located next to the borehole seismic stations. Wellhead (blue dot) and horizontal boreholes (blue lines) are shown. The inset map shows the hydraulic fracturing area (red square) in Poland. The map was created using the free software GMT Version 4.5.16 Released (https://www.soest.hawaii.edu/gmt/) and finished with the free software LibreOffice Version 4.3.3.2 Released (https://www.libreoffice.org).

In recent years, the interest in the assessment and mitigation of the environmental impacts of HF has increased in some European countries. In this framework, the SHEER project (www.sheerproject.eu) aims to develop best practices for assessing and mitigating the environmental impacts of shale gas exploration and exploitation. A core activity of the SHEER project was the installation and maintenance of a dedicated monitoring system at an HF operational site at Wysin, NE Poland (Fig. 1). The monitoring aimed to collect comprehensive information on seismicity, changes of the groundwater and air quality, ground deformations and operational data. This work focuses on the assessment of the seismic response to HF operations, for one of the first full-scale HF stimulations in Europe and the first one, where a dense, dedicated multidisciplinary monitoring was set up in advance.

As part of the preparatory work, recent works analysed the background noise conditions at the Wysin network^26^. Such noise analysis, combined with the forward simulation of synthetic seismograms for realistic induced seismic sources, allowed to assess and map the monitoring performance at Wysin before the beginning of HF operations. According to those results, all seismicity close to the injection wells above a magnitude of completeness of M_w_ 0.10 to 0.45 during night and day hours respectively, is expected to be registered. In this work, the seismic response of HF stimulations at Wysin are analysed and discussed, over a 4-month period involving different stages before, during and after the ending of HF stimulations. The discussion on short-term impacts of HF expands on the results from the air quality and groundwater monitoring.

## Geological Conditions, HF Operations and Monitoring System at Wysin

The target shale gas exploration and exploitation site at Wysin, in the central-western part of the Peribaltic Syneclise of Pomerania, NE Poland, is located within the Baltic Basin, which underlies much of the northern margin of the country as well as extending north under the Baltic Sea (Fig. S1). The Baltic Basin has a simple geological structure that is relatively undeformed tectonically. It contains a sequence of Palaeozoic to Mesozoic deposits, including Lower Palaeozoic organic-rich marine shales that are prospective for shale gas and oil development^27^. The geological sequence includes Cambrian sandstones and shales at a depth of approximately 4 km below ground level, overlain by Ordovician marly limestone, mudstone and siltstone and Silurian shales interbedded with dolomitic limestones. Much of the pre-drilling understanding of the regional and local geology is derived from the studies into the environment and shale gas exploration produced by the Polish Geological Institute (PIG-PIB) and associated organisations^28,29^. Previous drilling log of research boreholes close to the Wysin site, such as Koscierzyna IG-1 (8.25 km away, Fig. S1), provided information on the local lithology and stratigraphy (Table S1). Velocity models derived from Koscierzyna IG-1 are consistent with high-resolution 3-D seismic model for Poland at the location of the Wysin site^30^ (Fig. S2). The closest fault is located relatively far from the HF area, about 15 km NE from the wellhead, striking NW-SE^31^, which may not incur any effect on the structure of the rocks in the vicinity of the Wysin site (Fig. S1). However, we note that the 2D seismic profiles carried out during pre-operational surveys^29^ revealed parallel fault structures to the main fault (NW-SE) about 5 km away of the wellhead towards NE and SW (Fig. S1b).

HF operations were carried out along two horizontal boreholes, named Wysin-2H and Wysin-3H during 10 days each (2016, June 9–18 and July 20–29, respectively). HF boreholes are located at about 4 km depth and oriented WNW-ESE, with approximate horizontal lengths of 1.7 km each. According to the information provided by Polish Oil and Gas Company (PGNiG), the HF stimulations were divided in 11 injection stages for each horizontal HF borehole, reaching a total volume of 18812 m^3^ and 17230 m^3^ for the two stimulations (Wysin-2H and Wysin-3H) respectively, and maximum pressures at the well head between 84.3 and 90.5 MPa (PGNiG report by the support department of Geological Work in 2016). The experiment at the Wysin site implemented a dedicated multidisciplinary monitoring (Fig. 1) to jointly assess for the first time in Europe the short- and long-term risk connected to the most relevant potential hazards of HF operations: induced seismicity, air pollution and groundwater contamination.

The seismic monitoring includes a distributed network of 6 broadband stations, 3 small-scale arrays, each composed of 8 to 9 short-period stations, and 3 shallow borehole stations^26^. A hybrid and flexible seismic monitoring system was planned to identify and characterize the whole spectra of seismic consequences of HF operations. Broadband sensors with a sampling rate of 200 Hz provide reliable waveform recordings over a broad range of frequencies, allowing to analyse weak to moderate seismicity taking place in the local environment, at least up to 10 km distance from the operational well. On the other hand, a surface short period seismic installation benefits from the arrangement of the sensor geometry in multiple arrays. Surface arrays with a sampling rate of 500 Hz aim to detect, locate and characterise weak microseismic events, including those directly associated to hydraulic fracturing and help to track the migration of the fracture process in the vicinity (max 500 m distance) from the HF boreholes. In addition, the detection performance of weak events is improved by shallow underground seismic installation, within monitoring boreholes, since underground sensors are less affected by seismic noise; at the Wysin site, the shallow boreholes installation at depths of ~50 m could only partially reduce the seismic noise^26^. The monitoring network was fully operational from November 2015 to January 2017, allowing for continuous recording during the pre-, co- and post-operational phases. The seismic monitoring is combined with independent monitoring of air and water conditions, which help to track the environmental footprint of HF operations.

The air quality was monitored by an automatic air pollution monitoring station at Stary Wiec village, about 1100 meters east of the wellhead (Fig. 1). The station location was chosen in order to detect and investigate the possible impact of shale gas extraction related activities on the air quality in the surrounding inhabited areas and considering the prevailing, eastward wind direction. Natural gas extraction procedures can affect the quality of surrounding air at all stages in various aspects^32^. In the case of uncontrolled, massive methane outflows from the installation, e.g. Aliso Canyon blowout case, ambient methane levels can reach tens of ppm at a distance of kilometers from the source^33^. To take into account the above mentioned possibilities the station was equipped with a standard set of analysers of gaseous and particulate air pollutants, a meteorological module and additionally, a set of carbon dioxide, methane, non-methane hydrocarbons and radon concentration sensors. The measurements covered the period from July 2015 to July 2017, thus enabling background levels of air pollutants to be determined before, during and after the HF took place, as well as during the well closure operations. Data has been collected as 1-min averages, what allowed to identify fast changes and short duration anomalies of pollutant levels coming from close sources, e.g. from the well area.

The groundwater monitoring network consists of four boreholes (GW1 to GW4; Fig. 1), in which a downhole probe (CTD-Divers, Schlumberger) was installed at mid-point of the screened interval in December 2015. They record absolute pressure, temperature and specific conductivity every 15 minutes. Since the probes are non-vented, the installation is completed by a barometric probe (Baro-Diver, Schlumberger), measuring the atmospheric pressure and air temperature. The pressure sensors in GW1, GW3 and GW4 have a depth range of 100 m H_2_O with an accuracy of ±5 cm and a resolution of 2 cm. The GW2 pressure sensor has a depth range of 50 m H_2_O with an accuracy of ±2.5 cm and a resolution of 1 cm. The atmospheric pressure sensor has an accuracy of ±0.5 cm and a resolution of 0.2 cm. Specifications for temperature and conductivity sensors are the same for all probes. The temperature is measured with an accuracy of ±0.1 °C and a resolution of 0.01 °C. Accuracy and resolution are respectively ±1% and 0.1% of the reading for the electrical conductivity. The absolute pressure recorded by the sensor is converted to water levels in meter above sea level (m.a.s.l.) by subtracting the atmospheric pressure (from the Baro-Diver), and knowing the elevation of the well and the depth of the probe (see additional information^34^).

## Results

### Shallow artificial seismic noise sources

The operational data, provided by PGNiG, includes the total injected volume, pressure and perforation depth for each stage, but no accurate timing for the start and end of injection operations. However, all borehole stations recorded significant temporal anomalies in the noise amplitude during all days of HF operations. No significant increase on the seismic noise was detected at other, more distant, surface stations. The Seismic Noise Amplitude Increase (SNAI) can be clearly identified for all treatment days (Fig. S1). The SNAI duration is estimated by a spectral analysis (Method M1), revealing a good correlation with the injected volumes (Fig. S2); furthermore, a common spectral pattern of all SNAI signals reflects their common origin.

**Figure 2:**
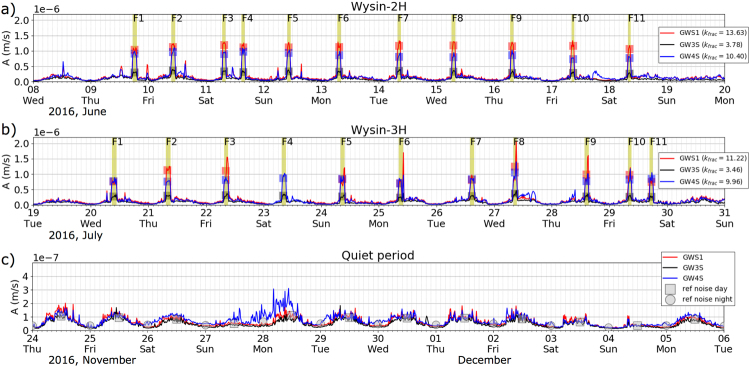
Average absolute amplitude of seismic signals is calculated every 15 min at borehole stations, applying a bandpass filter between 2 and 15 Hz. Three different time intervals of 12 days are considered: (**a**) HF stimulations at Wysin-2H, (**b**) HF stimulations at Wysin-3H and (**c**) quiet period after the end of all HF operations. Amplitude is normalized to velocity units removing the instrument response. Yellow bands indicate the SNAI duration associated with the frac stages (F1 to F11). Red, black and blue squares show the average amplitude during each frac stage for the borehole stations GWS1, GW3S and GW4S respectively. SNAI ratios (k_frac_) for each sensor is shown in the legend (see Method M2).The average amplitudes according the diurnal variation between 6:00 and 18:00 h are shown for day hours (gray squares) and night hours (gray circles). Time marks are at 2-hr intervals.

SNAIs accompanying each HF stage are analysed to assess the distribution of amplitude increase with respect to a reference baseline, extracted from a quiet period, to understand and locate their source (Fig. 2). With this aim, three different time intervals of 12 days were considered: one including all HF stimulations at Wysin-2H (June 8–20, 2016), a second one for HF stimulations at Wysin-3H (July 19–31, 2016) and a quiet period after the end of all HF operations, when the industrial installation was completely removed (November 24 - December 6, 2016). The average absolute amplitude of seismic signals is calculated every 15 min at borehole stations, applying a bandpass filter between 2 and 15 Hz, which corresponds to the frequency range mostly affected by the SNAI. The amplitude is normalised to velocity units removing the instrument response in order to compare results from different borehole sensors. Each HF stage is clearly identified by SNAIs (yellow bands in Fig. 2a, b), where the amplitudes experienced a significant increase over period of 1.5 to 2 h. Other shorter amplitude anomalies (durations of less than 1 h) can also be detected close to some HF stage sources (e.g. F1, F2 and F5 in Fig. 2a), possibly reflecting other anthropogenic noise. Similar, natural daily background noise oscillations are exhibited for all the three time periods; even a decrease of the daily noise during weekends can be appreciated (Fig. 2c). Generally, the amplitudes of the SNAI remain constant with small variations for different HF stages; in some cases, the amplitudes show an increase throughout single HF stages, with larger noise amplitudes at the end of a stage (e.g. F8 and F9 in Fig. 2b), possibly due to an overlap of multiple industrial activities or higher flow/injection rate. The ratio (k_frac_) of the average amplitude during SNAI (hereafter referred as SNAI amplitude) with respect to a reference baseline changes at different sensors, but remains constant over each HF stimulation (Figs 2 and S3, and Method M2). We observe small variations of k_frac_ between the HF at the two wells: for example, k_frac_ is always largest at sensor GWS1, but decreases from the stimulation of Wysin 2 H (k_frac_ 13.63) to the stimulation of Wysin 3 H (k_frac_ 11.22), while k_frac_ at other sensors experience a smaller change. These variations imply a small change of the locations of the anthropogenic noise sources, which were active during the two HF stimulations. Finally, SNAI amplitudes, injected volumes and maximum pressures show no clear correlation.

**Figure 3:**
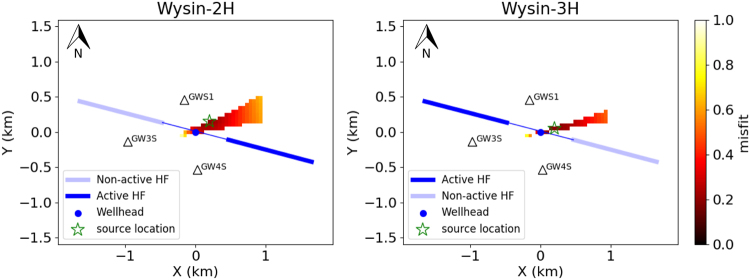
Location of SNAI (green open stars) through the modelling of amplitude decay during the HF stimulations at Wysin-2H (left) and Wysin-3H (right). Borehole stations are shown with black open triangles. We only assess the misfit in those grid points for which we observe the following amplitude relation: A_GWS1_ > A_GW4S_ > A_GW3S_ (Method M3).

Classical location methodologies of picking arrival times cannot be applied to locate the SNAI, so alternative amplitude-based methods were used, similar to those used in volcano environments for non-impulsive signals^35^. An approach fitting the decay of SNAI amplitudes as a function of the distance to the source, according to the geometrical spreading (Fig. 3 and Method M3), was implemented to locate the SNAI source. During the Wysin-2H stimulation, the noise source is located 250 m NE from the wellhead, while during the Wysin-3H stimulation, the source is 210 m ENE from the wellhead. The seismic noise source is thus not at the depth of the HF, but located at the surface in the vicinity of the wellhead. The resolved location of the noise source also explains the observation of SNAIs only at shallow boreholes, which are located much closer (<1 km) to the wellhead, with respect to the other surface stations. The source location and spectral characterisation of SNAI signals suggest they correspond to artificial shallow sources active during HF operations, such as vibrations excited by the pump trucks.

**Figure 4:**
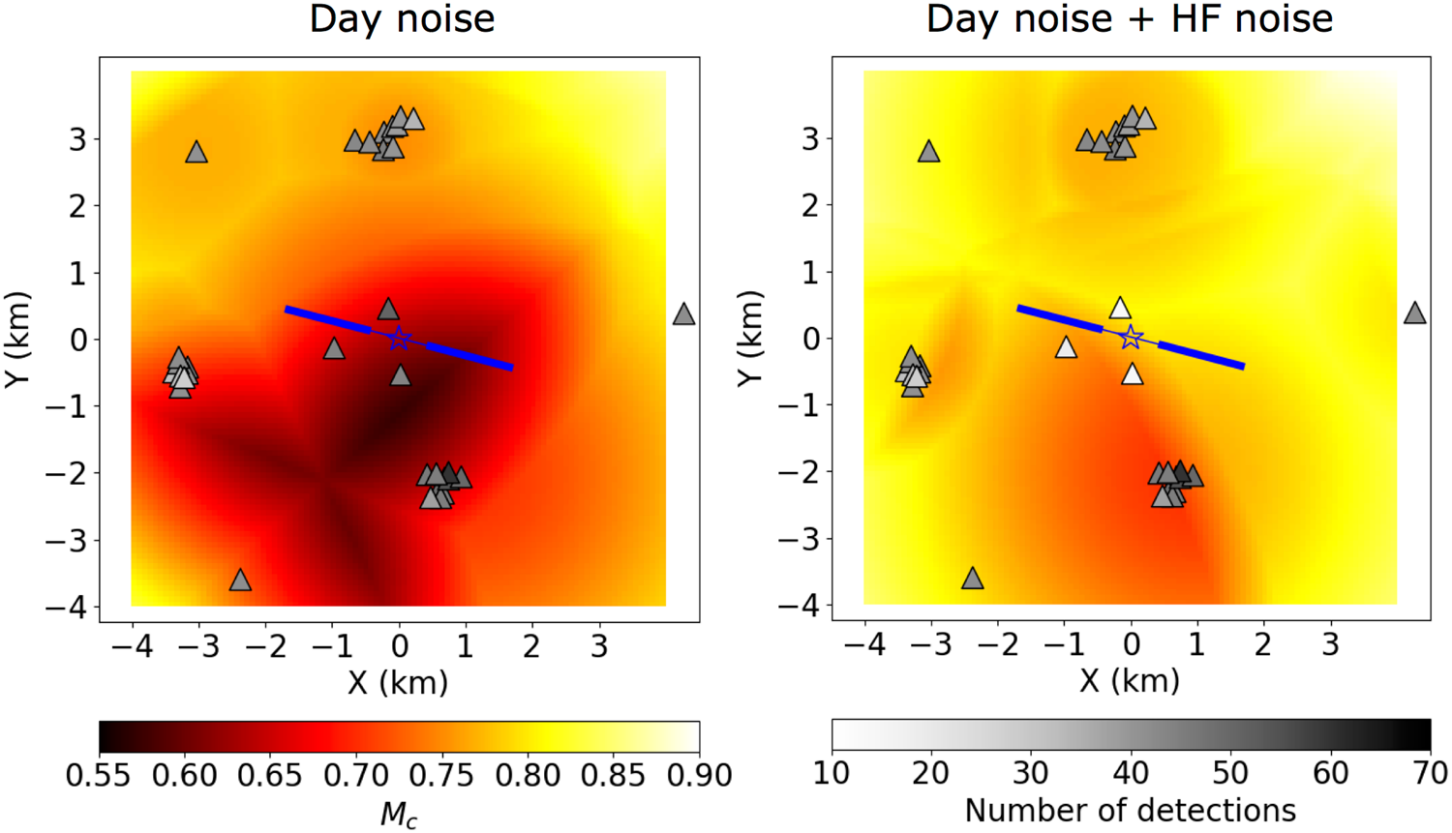
Spatial monitoring performance at Wysin site in terms of magnitude of completeness using an amplitude threshold approach estimated from noise recording before HF operations during day hours (left) and, in addition, considering the noise amplitude increase during the HF operations (right). Grey color scale identifies the number of synthetic events detected for each station (see technical details^26^). Star shows the HF area (vertical drilling) and blue lines indicate the horizontal HF drillings.

The SNAI sources strongly contaminate the seismic signals of shallow borehole stations, which are closest to the HF wells, and thus temporarily reduce the network detection performance of weak induced events during HF operations. In a previous work, the monitoring performance was assessed in terms of the magnitude of completeness (M_c_) at the Wysin site using noise reference levels from recording data before HF operations^26^. To account for the changed noise conditions, the M_c_ is recalculated according to the estimated SNAI ratios (Figs 2 and S3, and Method M2) following the same approach described in previous works^26^. The noise conditions change during day hours, because HF operations took place between 6:00 and 18:00 h only, increasing the M_c_ from 0.55 to 0.80 around the HF area (Fig. 4).

### Induced seismicity

Continuous seismic recording were processed at the Wysin site before, during and after the HF operations that took place on June and July 2016. During the seismic monitoring period, automatic event detection procedures were performed to assess the background seismicity and identify the seismic signals caused for the HF stimulations, which could be related to induced or triggered seismicity. The most relevant results are here shown for a 4-month period between June and September 2016. We apply a recently developed automated full waveform detection and location algorithm based on waveform stacking and coherence analysis (Method M4). This technique has been successfully applied in previous work, showing an improvement with respect to classical detection methods both for natural and induced seismicity^21,36^. Moreover, the detection algorithm performance at Wysin site, in terms of M_c_, was verified by processing a realistic synthetic dataset^26^.

**Figure 5:**
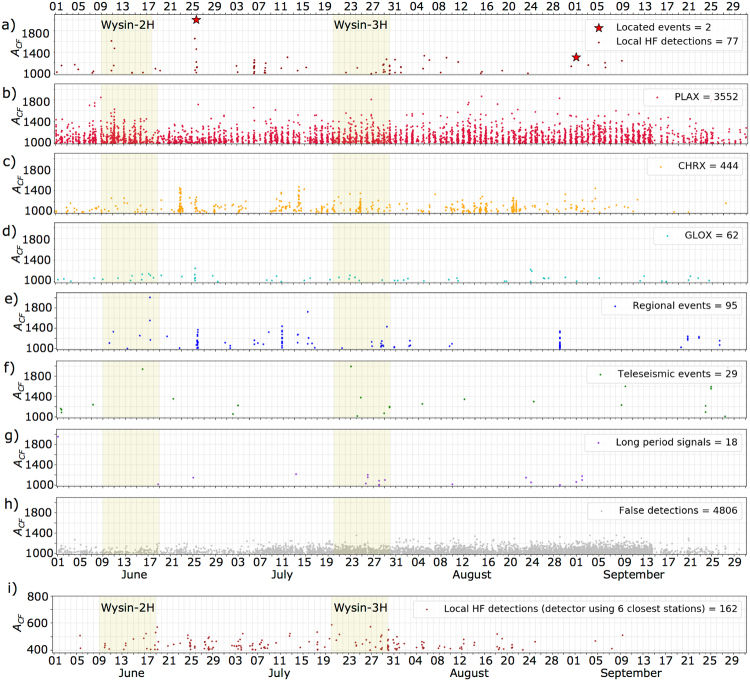
Detection and classification of seismic signals before, during and after the HF operations at the Wysin site. Each detection is identified by the time and the maximal coherence (A_cf_) obtained from Lassie detector (Method M4). The dataset has been classified manually according different categories (see legend in each box). Yellow bands indicate the 10-days period for the HF stimulations in Wysin-2H and Wysin-3H. Time marks are at 1-day intervals.

Automated detections have been manually revised and different types of seismic signals have been identified (Figs 5, S5 and S7), allowing the classification of signals into different categories. The seismic signals directly related to HF operations should arrive first at the borehole stations, which are the closest stations to the HF wells. Detections showing such a temporal pattern of arrival time will be referred hereafter as “local HF detections”. However, most of the local HF detections correspond to very weak events, which signals are only visible at the three operational borehole stations and often showing a low signal to noise ratio (Fig. S5b). Only two local HF detections were recorded at all other stations, allowing a robust hypocentral location, that will be discussed later (Fig. S5a). On the other hand, a large number of local events is classified as those detections recorded only at one of the seismic arrays (Fig. S5c). The array installed in Płachty (PLAX) shows the largest number of detections (3552), followed by the array in Chrósty (CHRX, 444 detections) and, in last instance, the array in Głodowo (GLOX, 62 detections). The PLAX array shows continuous local detections during the whole period, including pre-, co- and post-HF phases (Fig. 5b); a clear daily variation of the detection rate is observed, with the largest number of detections during night hours, between 20:00 and 3:00 h (Fig. S6), when the background seismic noise is minimum. The activity at the CHRX array is moderate (Fig. 5c). Short duration sharp increases of the detection rate are observed (e.g. June 23^rd^ and July 14^th^, 2016). One of such episodes is recorded during the Wysin-3H stimulation on July 24^th^, 2016, but signals are not visible at borehole stations; since this episode occurs in the period between two frac stages (Fig. S7c), when the borehole stations records are not affected by SNAI, it can be excluded that it originated in the vicinity of the HF operations. The GLOX array shows the lowest activity, with a few isolated detections (Fig. 5d). Since these events are not recorded at the shallow borehole stations, they reflect very weak events from local natural or anthropogenic sources close to the villages, where the arrays were installed, and cannot be associated with the HF operations. Although the detector algorithm is tuned to only reveal events originating in a local seismogenic volume (Method M4), a few regional and teleseismic events are still detected, which appear at all seismic stations, but with a pattern of arrival times which corresponds to a source located at a far distance from the HF area (Fig. S5d,e). Different classes of regional events are recognised, depending on the backazimuth revealed by the Wysin network. Three regional event sequences are identified on June 25^th^, July 10^th^ and August 29^th^, 2016, and a small one during the Wysin-3H stimulation on July 28^th^, 2016. A few, weak, long period (LP) signals are detected, with dominant frequencies of 3 to 5 Hz, observed at several stations (Fig. S5f), which cannot be localised. A number of false detections are also identified; their rate varies over time, mostly in consequence of the number of operational stations (Fig. 5h). In an attempt to improve the detection of weak events close to the HF wells, a second detection was run, using 6 closest stations (3 boreholes and 3 surface stations, one for each array). The number of local HF detections increase from 77 to 162 events (Fig. 5i and Method M4). These local HF detections are identified until beginning of September, roughly corresponding to the end of the HF operations and industrial activities. No local HF detection corresponds to the frac stages (Fig. S7a,i), probably because of the SNAI influence, but some happens shortly after these operation stages.

**Figure 6:**
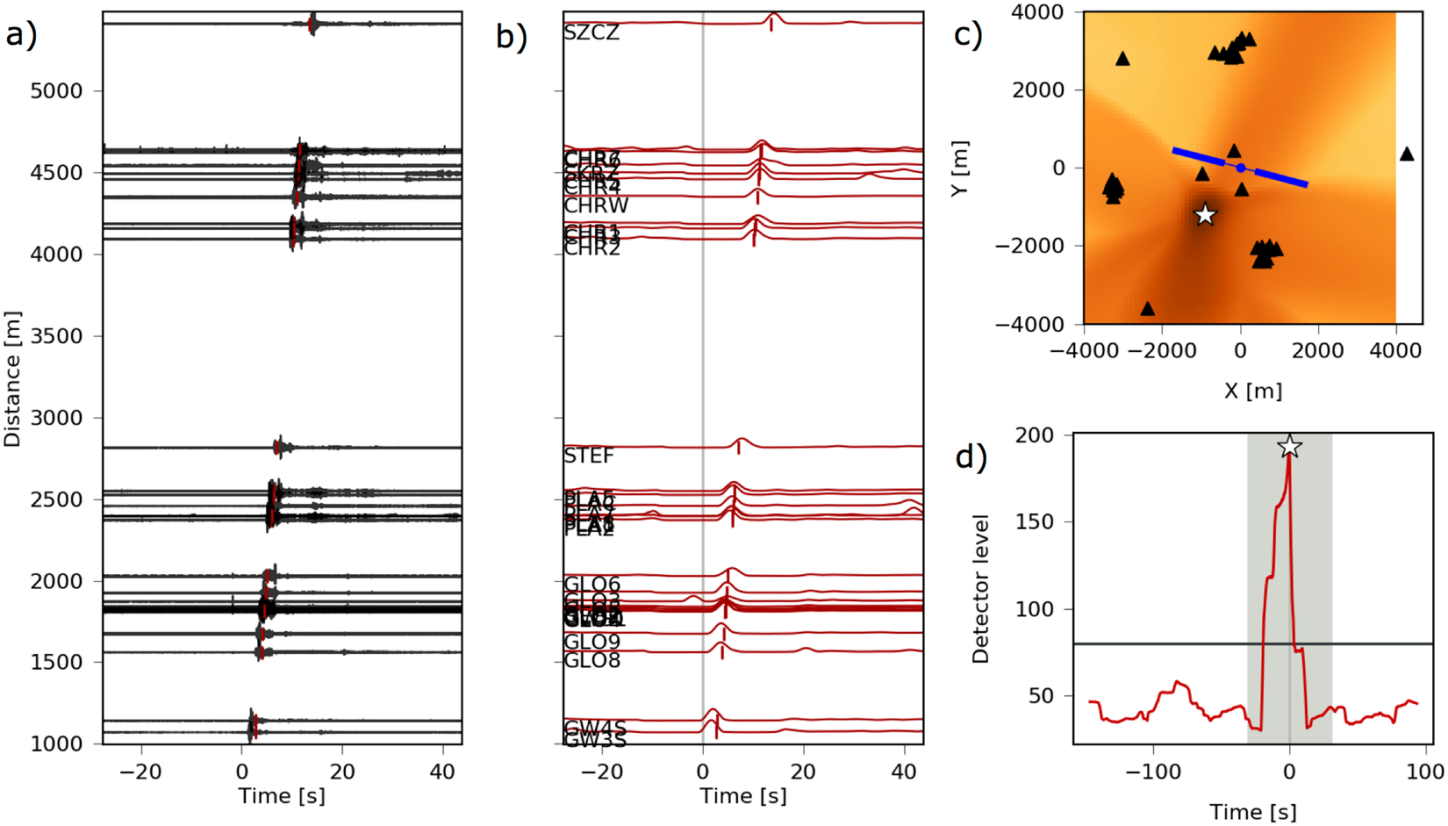
Hypocentral location on 2016 June 25^th^, 20:08:26 UTC time (Method M5). (**a**) Waveforms sorted by hypocentral distance. (**b**) Characteristic function (normalized amplitude envelopes) for each trace. These are used for travel–time stacking corrected with S-wave speed (red lines). The markers indicate the (best fit) synthetic arrival time of the S-phase at each sensor. (**c**) Coherence (stack) map for the search region. Dark colors denote high coherence values. A white star marks the location of the detected event. Sensor locations are shown with black triangles. (**d**) Global detector level function in a processing time window from −20 to 20 s around the origin time of the detected event. The cutout time window used for the coherence map is shown in gray color. White star indicates the detection exceeding a detector level threshold of 80.

**Figure 7:**
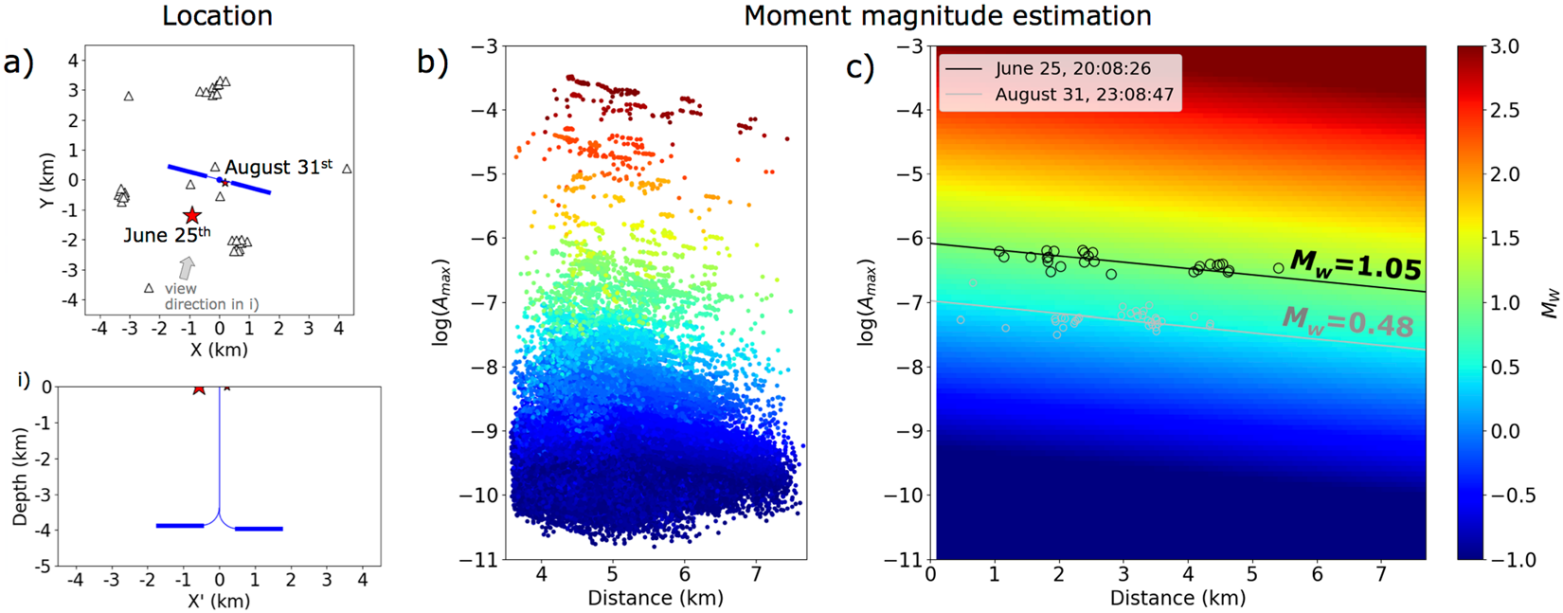
Location and moment magnitude (M_w_) estimation for the two main weak events detected at the Wysin site (Method M6). (**a**) Map view (top) and depth section (down) displaying the located events (red stars). Stars are scaled according the M_w_ estimation in (**c**). Triangles show seismic stations. HF boreholes are indicated with blue lines. (**b**) Maximum amplitudes for each source and each station plotted against hypocentral distance for the complete synthetic catalogue generated in previous work^26^. (**c**) M_w_ estimation using a domain extrapolation defined from the microseismic synthetic catalogue in (**b**). Black and gray dots show maximum amplitudes observed at all seismic stations for the two events displayed in (**a**). Black and grey lines represent the Mw estimation for both events (see legend).

In conclusion, while realizing an effective M_c_ ~ 0.8 during the frac stages, only two significant events could be assigned to the volume potentially affected by the HF operations. They are recorded by most stations and the signal quality allow a robust location. Both events have epicentral locations close to the HF site, but they have very shallow depths (<150 m), much shallower than the HF horizontal wells. The seismic signals are dominated by high frequency surface waves, propagating with a velocity of ~400 m/s (Figs 6, 7a and S8, Method M5), consistent with expected near-surface shear-wave velocities in the uppermost 5 m^37,38^. The location results has a better resolution and show a sharper coherence peak for the June 25^th^, 2016 event (Fig. 6c,d), with respect to the August 31^st^, 2016 event (Fig. S8c,d), what reflects their different magnitude and the quality of recordings (Fig. S9). The first event is located 1500 m SSW of the wellhead; the second one is located closer to the HF area, just 220 m ESE of the wellhead. The magnitudes (Method M6) of both events are successfully estimated to be M_w_ 1.05 ± 0.07 and M_w_ 0.48 ± 0.09 (Fig. 7), taking advantage of the synthetic seismogram database computed for a range of locations, depths and moment magnitudes^26^.

### Multidisciplinary monitoring

**Figure 8:**
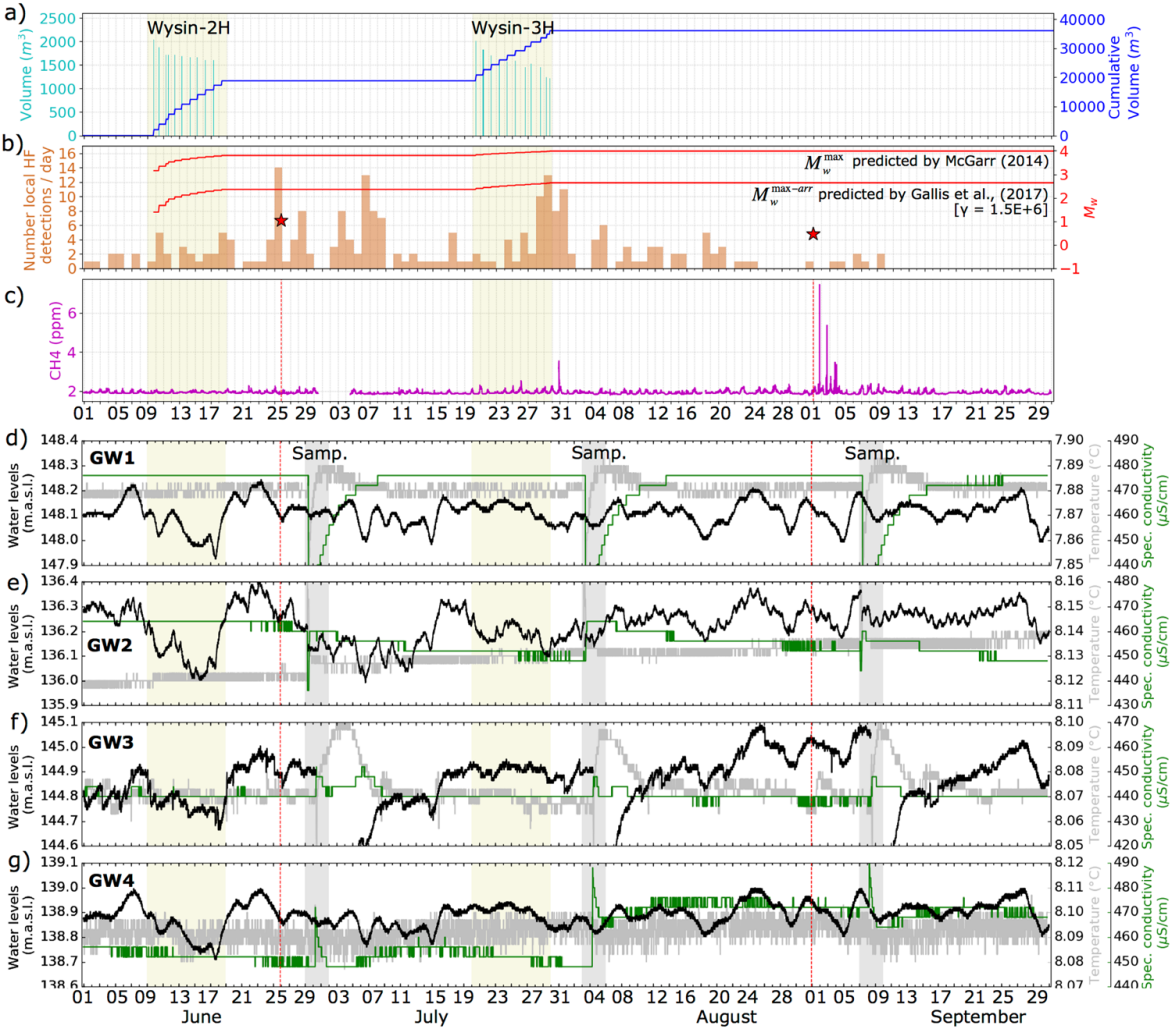
Correlation among fluid volumes injected, seismicity, air pollution and groundwater conditions for 4-month period involving different stages before, during and after the termination of HF stimulations. Yellow bands indicate the 10-days period for the HF stimulations at the Wysin-2H and Wysin-3H. Time marks are at 1-day intervals. (**a**) Fluid volumes injected in each frac stage and the cumulative volume. (**b**) Distribution of local HF detections per day (left axis) and the located events with M_w_ (red stars, right axis). The maximum magnitude^39,40^ is also shown (red line). (**c**) Methane content (CH_4_) in ppm. (**d**–**g**) Water levels, temperature and specific conductivity (black, gray and green line, respectively) for each groundwater borehole. Note the same height is shown in y-axes for the water levels (0.5 m), temperature (0.05 °C) and specific conductivity (50 μS/cm). Gray bands (so-called Samp.) indicate groundwater-sampling periods where any changes of the groundwater parameters result from groundwater sampling. Vertical red dashed lines in (**c**) to (**g**) indicate the time of the largest seismic events according the red stars in (**b**).

Given the availability of simultaneous water and air monitoring, possible correlations between the production stages, observed microseismicity and changes in water and air parameters are investigated (Fig. 8). The air monitoring is here only discussed in terms of methane levels; water level, temperature and specific conductivity time series at four stations are also discussed from the groundwater monitoring. We focus on specific time periods. First, we consider 10 days intervals around the HF stimulations at the Wysin-2H and Wysin-3H (Fig. S10), to judge short-term changes in air and water conditions with HF operations. The air monitoring shows the occurrence of repeated anomalies of methane, lasting for one to several hours, exceeding the natural cycle of daily variation of these pollutants. A first anomaly is seen on July 30^th^ (Fig. 8c), shortly after the end of the second stimulation (Wysin-3H). The methane concentration reached 3.5 ppm, almost double of the average level of ~1.9 ppm. Finally, we focus on shorter time periods, when largest seismic events have been detected (Fig. S11), to investigate a potential correlation of seismic, air and water anomalies. A series of sharp, outstanding methane peaks of decreasing amplitude (maximum amplitude of 7.4 ppm) were recorded starting ~19 h after the occurrence of the M_w_ 0.5, August 31^st^ seismic event (Fig. 8c). No impact from HF activities was detected on the groundwater parameters at short- and medium-term scale (Figs 8, S10 and S11). The only visible changes in Fig. 8 result from groundwater sampling, during which water is pumped out of the boreholes.

## Discussion and Conclusions

The seismic response of one of the first real-scale HF experiment in Europe has been assessed by monitoring and analysing seismic records before, during and after the HF operations. Whereas the seismic noise characterisation in the pre-operational phase only depicted daily variations of the seismic noise amplitude, additional shallow artificial seismic noise sources at the wellhead are active during all HF stages for periods of 1.5–2 h, temporally reducing the signal-to-noise ratio (SNR) of local shallow borehole installations and increasing the M_c_ during day hours by ~0.25. The noise source could experience small shifts between the two wells stimulations according the recorded amplitude variation. It is assumed that the observed noise signals correspond to pumping trucks or other machinery involved in the fluid injection processes. The shallow borehole stations, which are closest to the HF site and should mostly contribute to detection and location of HF induced microseismicity, are those mostly affected by noise. A deeper borehole installation, where possible, should reduce the noise contamination by shallow noise sources and increase the amplitude of deeper HF induced microseismicity, leading to a substantial improvement of the SNR.

Given the accurate assessment of the monitoring conditions, it is concluded that the HF experiment at the Wysin site did not induce earthquakes with M_w_ > 1. We note that the Wysin site is tectonically inactive, lacking any background seismicity, encouraging the absence of microseismicity. Pre-operational surveys revealed parallel fault structures along NW-SE about 5 km away to the HF area (Fig. S1). The distribution and geometry of fault structures in the surrounding of injection sites can strongly affect the extent of induced seismicity. Our results do not reflect any activation of the mapped local faults, neither before the operations nor in consequence of HF. On the other hand, the maximal observed magnitude at Wysin is also in agreement with the M_w_ 4 empirical upper magnitude bound for injection induced seismicity^39^, and the physics-based prediction for the maximum size of arrested ruptures^40^, which yields a lower and more consistent value of M_w_ 2.6 (Fig. 8b and Fig. S14). The injected volume at the Wysin site is comparable to the HF cases of the WCSB^8^, which indeed triggered larger magnitude events of M_w_ ~ 4. However, at the WCSB, such seismicity has been interpreted as the result of local fault activation^41^, which did not occur at Wysin. Furthermore, the maximal magnitude detected at Wysin appears to be in good agreement with the recompilation of HF cases by Maxwell^3^ (blue circles in Fig. S14), which lists case studies spanning over a much broader range of injected volumes.

It has been recently observed at Fox Creek that only 10% of the pads and 15% of the wells in the Kaybob Duvernay are associated with seismicity, requiring a minimum injected volume to raise the seismicity rate to a sufficient level for observation, and suggesting that other geological factors play a prominent role in seismic productivity^42^. Geological information for different shale formations and fluid injection experiments has been compiled in the framework of other European projects (www.m4shalegas.eu*; openecho.jrc.ec.europa.eu*). However, there are still not conclusive results revealing a clear relation among geological formations and induced seismicity hazard^43^. The absence of detected microseismicity also agrees with the recent results for 53 wells fracked in the Guy-Greenbrier, Arkansas area^16^. There, half of the wells induced no detected seismicity above M_L_ 0, and only a few had events as large a M_L_ 1, and none with M_L_ > 3. Additionally, this reservoir formation have been stimulated by HF operations for first time during our target period and is characterized by a deep shale formation (~ 4 km depth), in comparison with 3.5 km in Kaybob Duvernay^44^, 2.5 km in US^13–15^ and 2.3–3 km in Sichuan Basin, China^12^.

We conclude that the adopted monitoring system, a relatively low cost and a combination of surface and shallow subsurface installation, proofed to be sufficient to detect and characterize significant induced seismicity (e.g. M_w_ 0.5 or larger) due to HF. The surface-monitoring concept is then successful for the detection of events relevant for most traffic light systems based on the maximum magnitude thresholds to limit the induced seismicity risk^45^. However, the detection capability are not sufficient to detect small fractures, track their migration, evaluate permeability changes, and ensure the integrity of bounding layers above and below the depth of injection. This target may be achieved through more expensive deeper installations, and 3D underground arrays.

The two shallow weak events with M_w_ 1.0 and 0.5 appear to be related with HF operations, although their shallow source indicates that they occurred very close to the surface, several kilometres above where the hydrofracs occurred. Both events are recorded days after the end of the injection. Such a delayed seismicity was also observed for other cases of triggered seismicity^16,41,46^. The largest event, took place at some distance (~1500 m) from the wellhead, whereas the second one is much closer to the region affected by HF operations. Although the detected events are weak, not exceeding magnitude M_w_ 1.0, no comparable natural seismicity has been observed in this area in the months preceding the operations. The spatial vicinity among the HF well and epicentres, and the temporal correlation between HF operations and seismicity occurrence, suggest a link between HF activities and these two events. Both events on June 25^th^ and August 31^st^, 2016, are very shallow, and the epicenter of the largest one even far from the region affected by hydraulic fracturing. Physical processes usually considered to explain triggered seismicity, such as stress perturbation or pore pressure change, are unlikely responsible for these small earthquakes, because these sources are too far from the injection zones and we have no evidence of a pore pressure connection from the wellbores depth to the surface. We also note the occurrence of a seismic sequence at regional distances taking place over the time of the largest event that could alternatively suggest a process of dynamic triggering for the M_w_ 1.0 event (Fig. 5a,e). Again, this hypothesis is unlikely since this event is very shallow and the perturbation small. On the other hand, the spatial location for the second event (M_w_ 0.5) very close to the wellhead suggests a link to human operations. The shallow depth and late occurrence (almost one month after the HF stimulation) may indicate the event could be related to operations carried out during the well disposal, rather than the fracking itself. Our requests for information from the operator about possible activities at the site went unanswered.

Observed short-term peaks in methane concentration in July and September 2016 differ significantly from mean values observed during these months (1.92 ± 0.27 ppm). These results are similar in magnitude to those measured during other campaigns in shale gas exploitation areas in the USA^47,48^, but no seismic correlation with air pollution effects were found. We note all these peaks were detected during wind conditions favourable for air pollution transport from the wells area to the air monitoring station, strengthening the hypothesis about a plausible source from industrial operations at the well head. The most significant anomaly recorded a maximum peak of 7.4 ppm for methane with a delay of hours after the M_w_ 0.5 seismic event, involving three peaks of decreasing amplitude in three consecutive days at almost the same time of the day (Fig. S11), suggesting some scheduled operation. These observations support our interpretation that the seismic event was induced by industrial activities associated with the post-operational well disposal, such as a mass shift or a strong vibration at the surface. However, we have not evidence to attribute both seismic and methane anomalies to the same operations at the well head because no repeated seismicity is detected and the delay between seismic event and methane is slightly large (~19 h) although both occur in less than one day. We also note other methane sources have not been identified in our target area at this time.

In terms of impact of HF on groundwater, short-term response to the seismic events could potentially occur as observed for weak, moderate, and large earthquakes (e.g. M ≥ 2.3)^49^. Recent works showed that three induced-seismic events in Oklahoma (M_w_ ≥ 5) affected the water levels at distances over 150 km from the epicentre^50^. Owing to the low magnitude of the detected events at the Wysin site, changes affecting water levels, electrical conductivity and temperature are expected to be of low amplitude, and occurring simultaneously or shortly after the seismic event. A few reasons for the absence of detected changes related to HF activities can be invoked. (1) The groundwater monitoring plan was designed to capture medium-term impacts. The equipment has lower resolution and precision than would be required to assess small short-term changes resulting from low magnitude seismicity. The temporal resolution (Δt = 15 min) might also not be optimal. Other authors studied the impact of low magnitude seismicity events (M_L_ < 1.5, epicentre at depth between 8 and 24 km) on groundwater levels using sensors with an accuracy of 0.1% and a resolution of 1 mm^51^. They concluded on the absence of significant rises or drops of groundwater levels. (2) The magnitude of the seismic events is very low although the equipment is located at a small distance from the epicentre (in comparison to previous studies^51^). (3) The semi-confined aquifer behaviour and the aquifer heterogeneities at the Wysin site^34^ are possibly less favourable to the detection of small changes^52^.

## Methods

This section includes a description of the following methods:

- M1: Spectral analysis and duration estimation of SNAI.
- M2: Estimation of the SNAI ratio.
- M3: Location of SNAI through the modelling of amplitude decay
- M4: Automated full waveform detection based on waveform stacking and coherence analysis.
- M5: Hypocentral location based on waveform stacking and coherence analysis.
- M6: Moment magnitude estimation using a microseismic synthetic catalogue.

### M1: Spectral analysis and duration estimation of SNAI

Spectrograms reveal an abrupt increase and decrease of the SNAI at frequencies between 2 and 80 Hz, which allows picking of the starting and ending time of the SNAI with an uncertainty of ~2 s (Fig. S1b). All SNAIs show a common peculiar pattern, where the first part of the signal (around 10 min) exhibits different frequency peaks to the subsequent signal (Fig. S1c,d). We have considered the duration of this pattern as a proxy for the period of HF operations. Under a constant flow, the total volume of injected fluid for each HF stage should correlate with the estimated HF duration. Fig. S2 shows the proportionality between injected volumes and HF durations, which confirms a clear correlation between injection parameters and duration of seismic noise anomalies. The volume of injected fluid decreases with the HF stage in each stimulation. However, we note that similar volumes were injected at Wysin-2H and Wysin-3H along different time periods, with a longer duration and, consequently, slower injection rates during the HF operations at the Wysin-3H.

### M2: Estimation of the SNAI ratio (k_frac_)

We estimate an average amplitude during each SNAI (so-called SNAI amplitude) at each borehole station; this value remains quite constant over each stimulation (Fig. S3). Consequently, similar SNAI amplitude variations are observed among borehole stations in each HF stimulation where a trend line is estimated for the SNAI amplitude (A_i_) in each borehole station, suggesting a common origin source for each stage of one HF stimulation. Note that some differences in the SNAI amplitude variations (e.g. F2, F3, F8 and F9 in Fig. S3b), could be related to the second amplitude increase at the end of some HF stages, as previously discussed (see section Results: Shallow artificial seismic noise sources). We extract a reference baseline (A_ref_) using the average amplitudes during day hours between 6:00 and 18:00 h from the quiet period (Fig. 2c); note that we take as reference, the daily background noise, because HF operations are always performed during day hours. Finally, we define the SNAI ratio for each HF stimulation as k_frac_ = A_i_/A_ref_. Thus, k_frac_ characterizes the amplitude increase observed in each borehole station with respect to background conditions.

### M3: Location of SNAI through the modelling of amplitude decay

Following similar approaches from volcano seismology, and driven by the lack of clear onsets in the noise signals, we use the amplitude of the signal to estimate the location of its source. We make the assumption of a radial symmetric radiation pattern, where the amplitude of the recorded signal (SNAI) is only dependent on the distance to the source, being controlled by the geometrical spreading. We perform a grid search for the noise source location, considering as a potential seismogenic volume the region 1 km^2^ around the wellhead. We only consider as potential locations those grid points for which the d_GWS1_ < d_GW4S_ < d_GW3S_, where d denote the distance from the grid point to a borehole station, considering that we observe the following amplitude relation: A_GWS1_ > A_GW4S_ > A_GW3S_. Next, for each potential grid point, we fit the scatter of amplitudes and distances by a geometrical spreading law *A* = *a/r*^53^, where *A* is the SNAI amplitude, *r* is the distance from the source to the receivers, and *a* is an attenuation constant depending on the material between source and receiver. A non-linear least squares technique is used to estimate the constant *a* and to assess the misfit for each grid point (Fig. S4). The source location is then estimated where the misfit is minimum (Fig. 3).

### M4: Automated full waveform detection based on waveform stacking and coherence analysis

We use an automated full waveform detection algorithms based on waveform stacking and coherence analysis, named Lassie (https://gitext.gfz-potsdam.de/heimann/lassie), to process the continuous seismic recordings at the Wysin site. Lassie calculates characteristic functions (CFs), which are based on the energy trace. The stacking for CFs at each seismic station is performed assuming a regular sparse grid of potential locations and origin times, providing a 4D matrix of coherence values. This information is used to build a time serie, where element corresponds to the maximum coherence of the 4D matrix for each time sample. A detection is then found when the time serie exceeds a threshold value. The spatial location associated to the coherence peak provides a first, rough location. At Wysin we consider a spatial grid of 4 × 4 × 2 km, and compute theoretical arrival times for a local crustal model^26^. Lassie was able to process 1 day of data in 5 h, on a single workstation (8 processors with 4 cores each one).

The choice of the detection threshold, later referred also as amplitude of the characteristic function (A_cf_), controls the detection performance: weak events can be detected at the cost of a larger number of false detections. We fix the threshold to 1000 (Fig. 5), following preliminary tests with synthetic data^26^. In a second application, using only the six closest stations (Figs 5i and S7i), we fixed the A_cf_ threshold to 400, by trial and error in order to limit the number of false detections.

### M5: Hypocentral location based on waveform stacking and coherence analysis

We use here the Lassie algorithm (Method M4) to improve the location accuracy, We extend and densify the spatial grid of potential locations, to a volume of 8 × 8 × 5 km. Synthetic arrival times for P and S phases are first estimated for a local crustal velocity model^26^. Since this model is unable to explain the arrival times at different stations, we test alternative homogenous velocity models with variable P and S wave velocities. Finally the best solution is found for shallow sources assuming a slow wave velocity of 400 m/s (Figs 6 and S9), which is reasonable for near-surface shear-wave velocities.

### M6: Moment magnitude estimation using a microseismic synthetic catalogue

The problem of magnitude estimation is not trivial, and important differences have been detected among different catalogues related to induced seismicity^54,55^. Furthermore, since different magnitude types and estimation techniques are used and transparent procedures to estimate magnitudes are not always provided, discrepant estimates may be given for the same earthquake. We apply a new approach to improve the M_w_ accuracy using a microseismic synthetic catalogue previously calculated. Synthetic waveform recording at each seismic station are generated for events within a predefined magnitude range coherent with the target weak events^26^. We can then plot the magnitude of each event, as a function of the hypocentral distance and the maximum amplitudes of synthetic seismograms recorded at each station (Fig. 7b). If a sufficient number of amplitude estimates is available, the full target domain can be then extrapolated, e.g. using a minimum search algorithm (Fig. 7c). The maximum amplitudes decrease with source-receiver distance due to geometrical spreading. This relation can be modelled for different magnitude values. Therefore, it is possible to retrieve the M_w_ for each of the two target events through a linear regression using the recorded maximal amplitudes and the source-receiver distances. We obtain M_w_ estimates of 1.05 ± 0.07 and 0.48 ± 0.09 for the June 25^th^ and August 31^st^, 2016 events, respectively (Fig. 7c).

## Electronic supplementary material

Supplementary Information
