# Supplementary material for: Induced seismicity response of hydraulic fracturing: results of a multidisciplinary monitoring at the Wysin site, Poland
Source: Sci Rep. 2018 Jun 5;8:8653. doi: 10.1038/s41598-018-26970-9 (PMC5988740; doi:10.1038/s41598-018-26970-9)
Supplement: Supplementary file 1 — Supplementary Information [file 41598_2018_26970_MOESM1_ESM.pdf]

Induced seismicity response of hydraulic fracturing: results of a multidisciplinary monitoring at the Wysin site, Poland

J. A. López-Comino[1,*], S. Cesca[1], J. Jarosławski[2], N. Montcoudiol[3], S. Heimann[1], T. Dahm[1], S. Lasocki[2], A. Gunning[4], P. Capuano[5] and W.L. Ellsworth[6]

[1] GFZ German Research Centre for Geosciences, Telegrafenberg, D-14473 Potsdam, Germany.

[2] Institute of Geophysics, Polish Academy of Sciences, ul. Ksiecia Janusza 64, PL-01-452 Warsaw, Poland

[3] School of Engineering, University of Glasgow, G12 8QQ Glasgow, United Kingdom

[4] RSKW Ltd, United Kingdom

[5] Dipartimento di Fisica, Università degli Studiy di Salerno, Italy

[6] School of Earth, Energy and Environmental Sciences, Stanford University (USA)

*Corresponding author: José Ángel López Comino (jalopez@gfz-potsdam.de)

Supplementary Information

Figures S1 - S14

Table S1

References

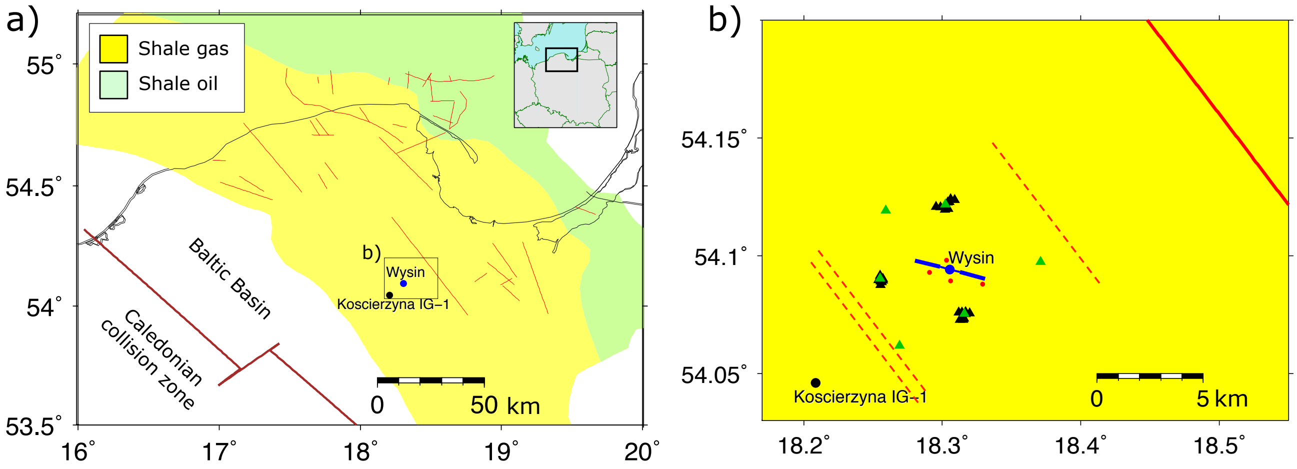

Figure S1. a) Structural-tectonic map of the Polish part of the Baltic region including the estimated area of resources of shale gas (yellow area) and shale oil (green area) in a model with thickness at least of 15 m of shale formation with Total Organic Carbon (TOC) contents > 2 % wt[1]. Fault structures (red lines) and the separation between the Caledonian collision zone and the Baltic Basin (brown line) are shown[1,2]. b) Zoom at the Wysin site showing the monitoring system and faults structure. Pre-operational surveys based on seismic profiles[3] revealed parallel faults structure (red dashed lines) to the closest main fault (red line). The monitoring system includes broad-band stations (green triangles), short-period stations (black triangles) and borehole stations (red circles). Horizontal fracking wells (blue lines) are shown. Wellhead of the Wysin site (blue dot) and Koscierzyna IG-1 research borehole (black dot) are also shown on both figures.

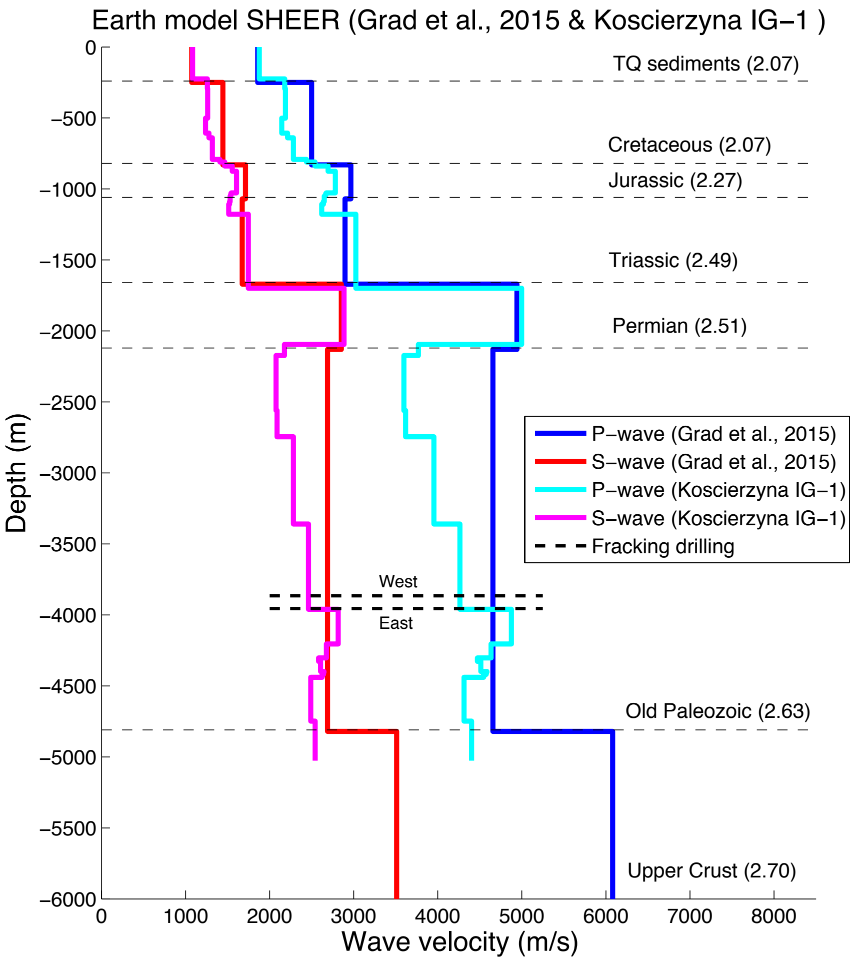

Figure S2. Comparison of local crustal model for the Wysin site according the high-resolution 3-D seismic model for Poland at the location of the fracking site[4] and the results from the Koscierzyna IG-1 research borehole located 8.25 km from the Wysin's wellhead. Each layer is identified with the name and the density value in g/cm^3 ([5]). The thick black dashed lines indicate the depth of the west and east horizontal fracking wells.

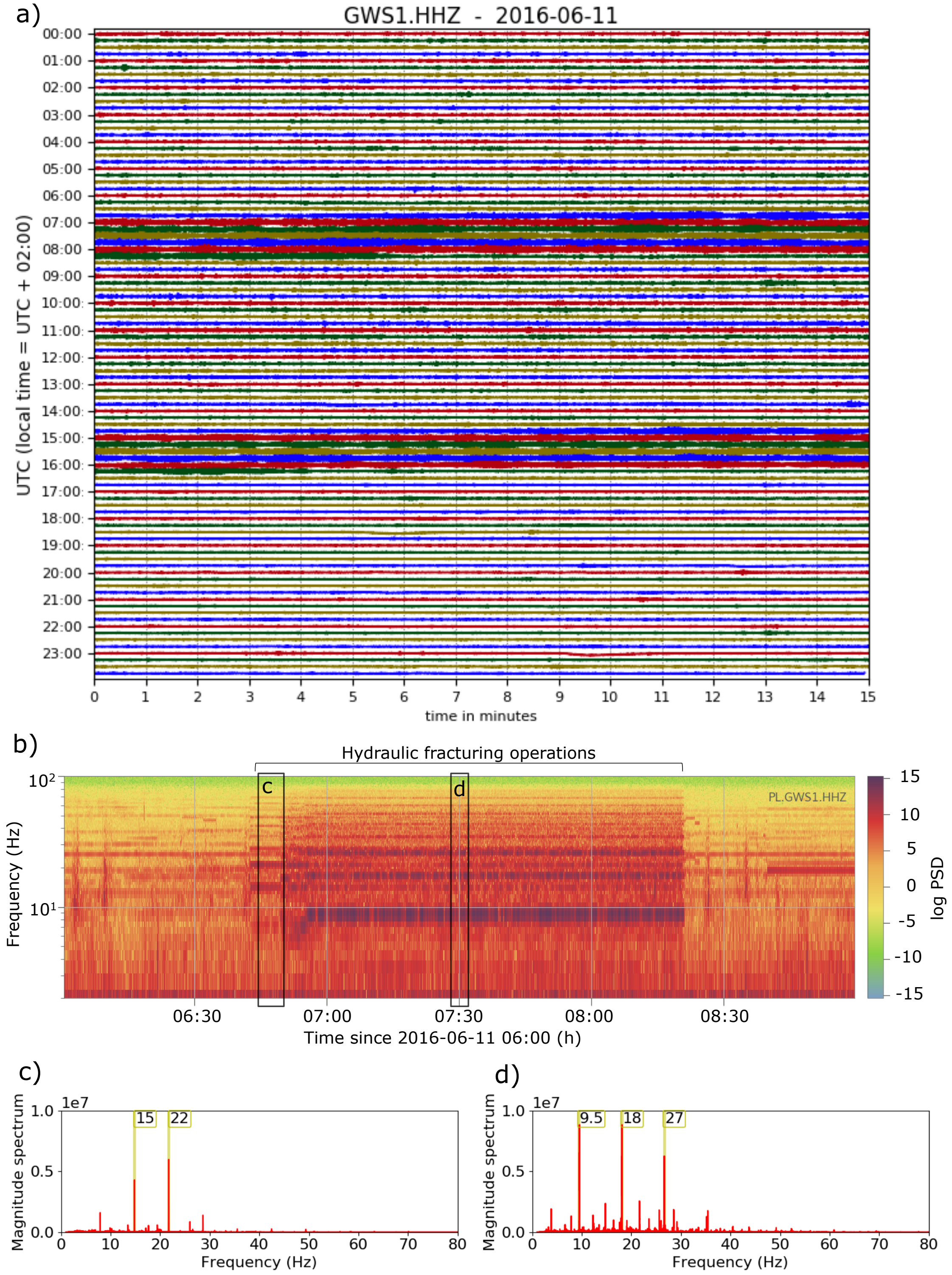

Figure S3. a) Seismograms from the borehole station GWS1 (vertical component) for a whole day (2016, June 11th). Time increases from top to bottom and from left to right. Time marks are at 1-min intervals, and each line is 15 min apart from the adjacent lines. Two frac stages are identified in different times (06:42:18 - 08:21:13 and 14:40:40 - 16:19:29). b) Spectrogram over 3 h (with 1s windows) for GWS1 during the first frac stage in this day. c) and d) Magnitude (absolute value) of the frequency spectrum over 10 min during the time intervals indicated in b). Yellow bands and boxes show the main peak frequencies and their values.

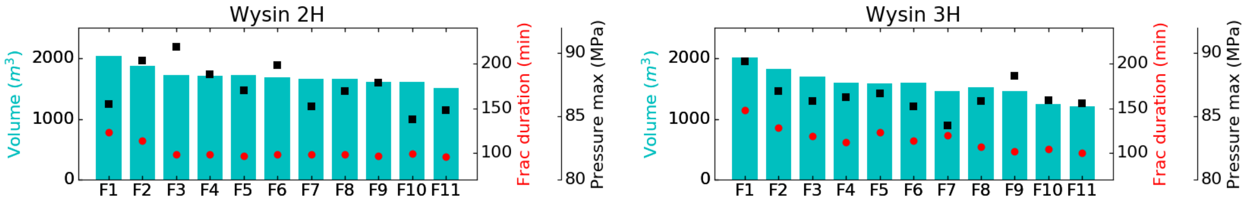

Figure S4. Total volume of injected fluid (cyan bars), SNAI duration (red dots) and maximum pressure (black squares) for each frac stage (F1 to F11). The results are shown for each horizontal HF drilling: Wysin-2H (left) and Wysin-3H (right).

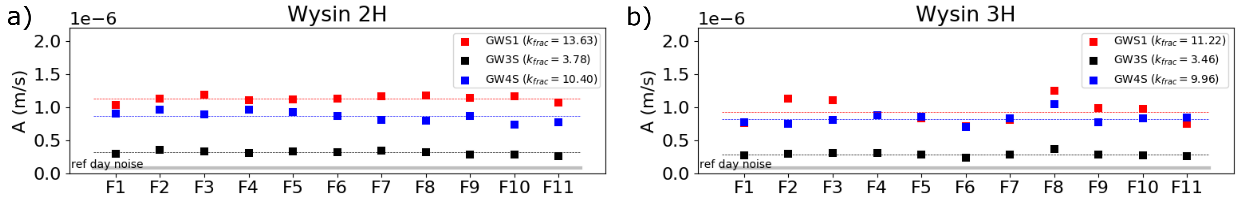

Figure S5. SNAI amplitudes in each borehole station for the different frac stages (F1 to F11) during HF stimulations at: a) Wysin-2H and b) Wysin-3H. We show a reference baseline using the average amplitudes during day hours between 6:00 and 18:00 h from a quiet period. Trend lines and SNAI ratios (k_{frac}) are estimated from the SNAI amplitudes in each borehole station (Method M2).

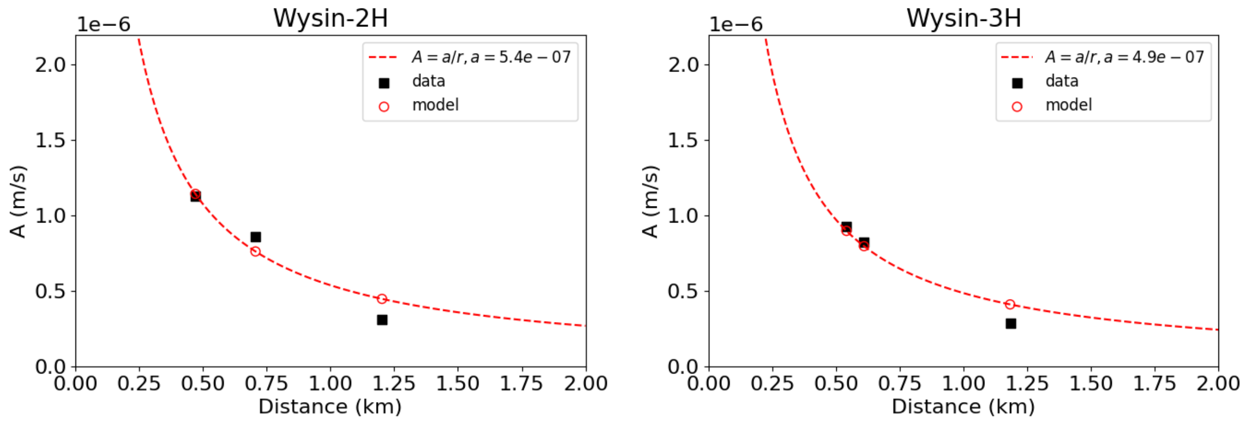

Figure S6. SNAI amplitudes recorded in each borehole stations (black squares) are fitted through a geometrical spreading law with attenuation constant, *a* (red dashed line). These adjustments correspond with the SNAI locations shown in Fig. 3 (Method M3).

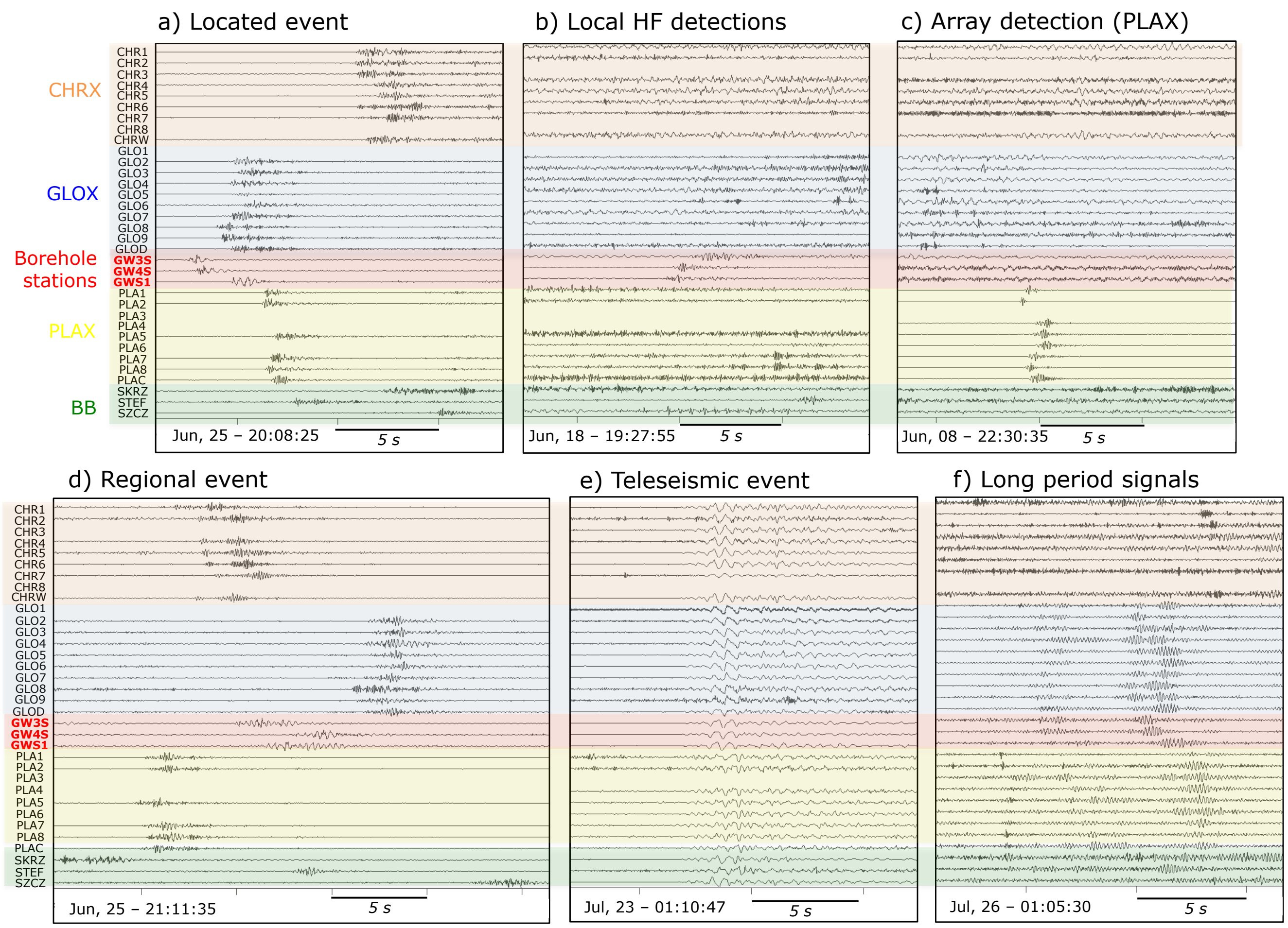

Figure S7. Examples of different kinds of detections (see top label for each box). Each trace shows the recorded signal for each seismic station. Waveforms are band-pass filtered in the frequency range 2–15 Hz. The time (s) is shown on the x-axis, and the reference time is displayed in the lower left corner of each box.

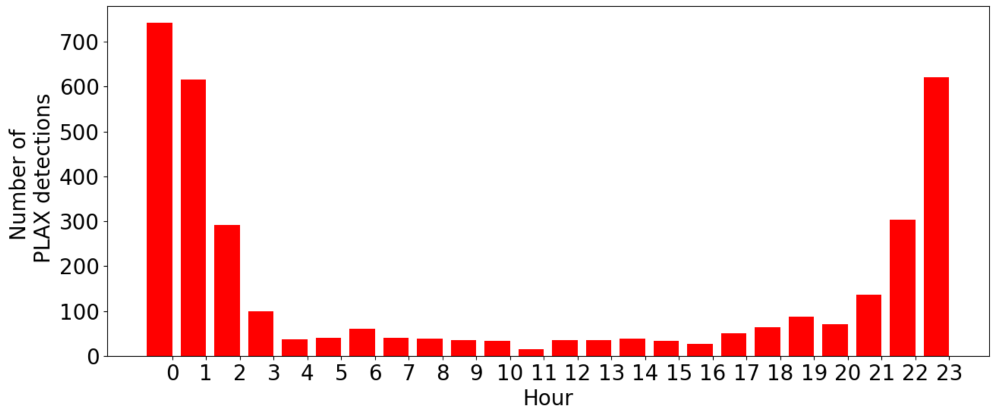

Figure S8. Diurnal variation of the number of local detections in the PLAX array. The x-axis shows the UTC hour (local time= UTC+2:00).

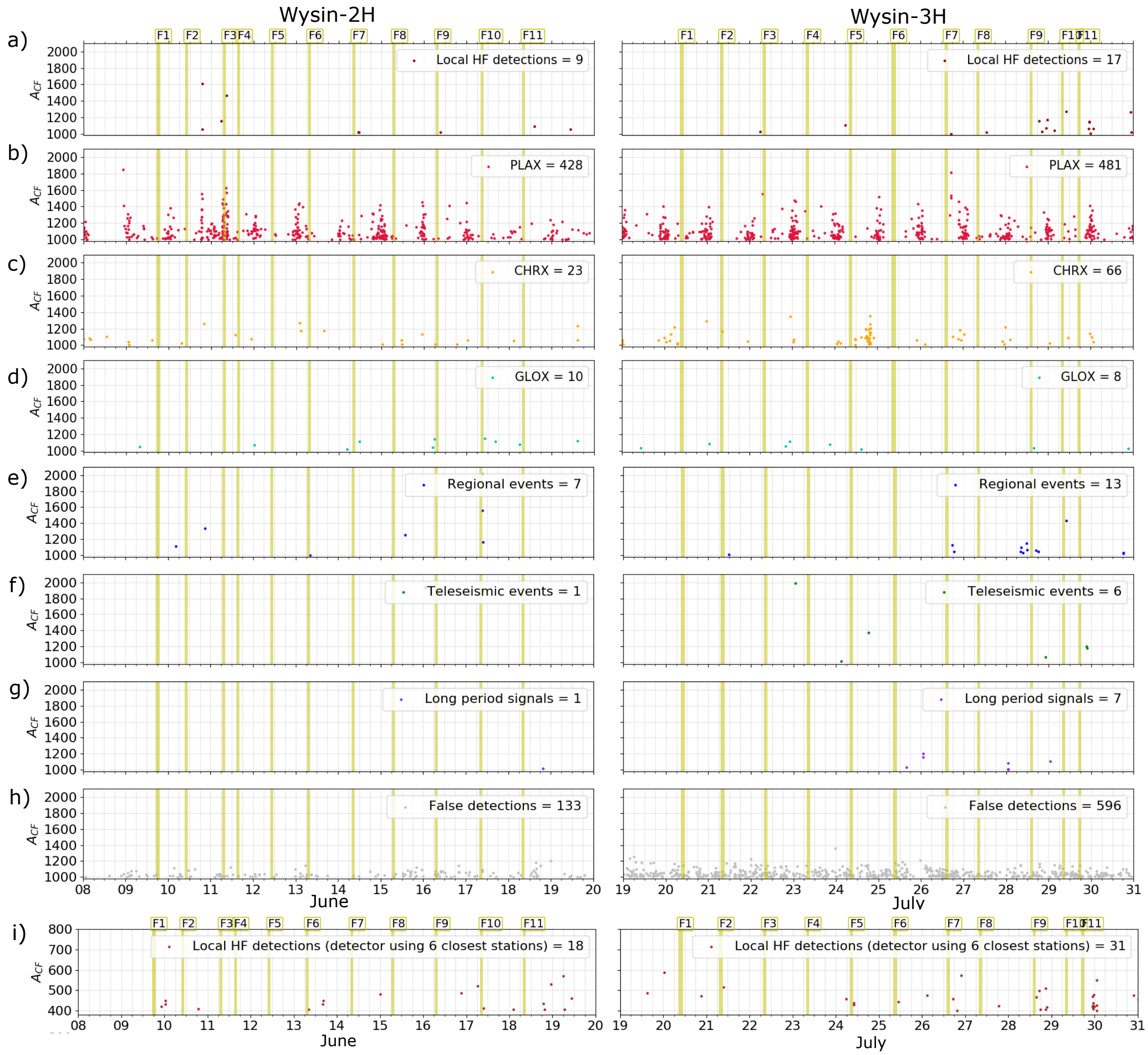

Figure S9. Zoom in Fig. 5 around the HF stimulations at Wysin-2H (left) and Wysin-3H (right). Each detection is identified by the time and the maximal coherence (A_{cf}) obtained from Lassie detector (Method M4). The dataset has been classified manually according different categories (see legend in each box). Yellow bands indicate the SNAI duration associated to each frac stage (F1 to F11). Time marks are at 6-hour intervals.

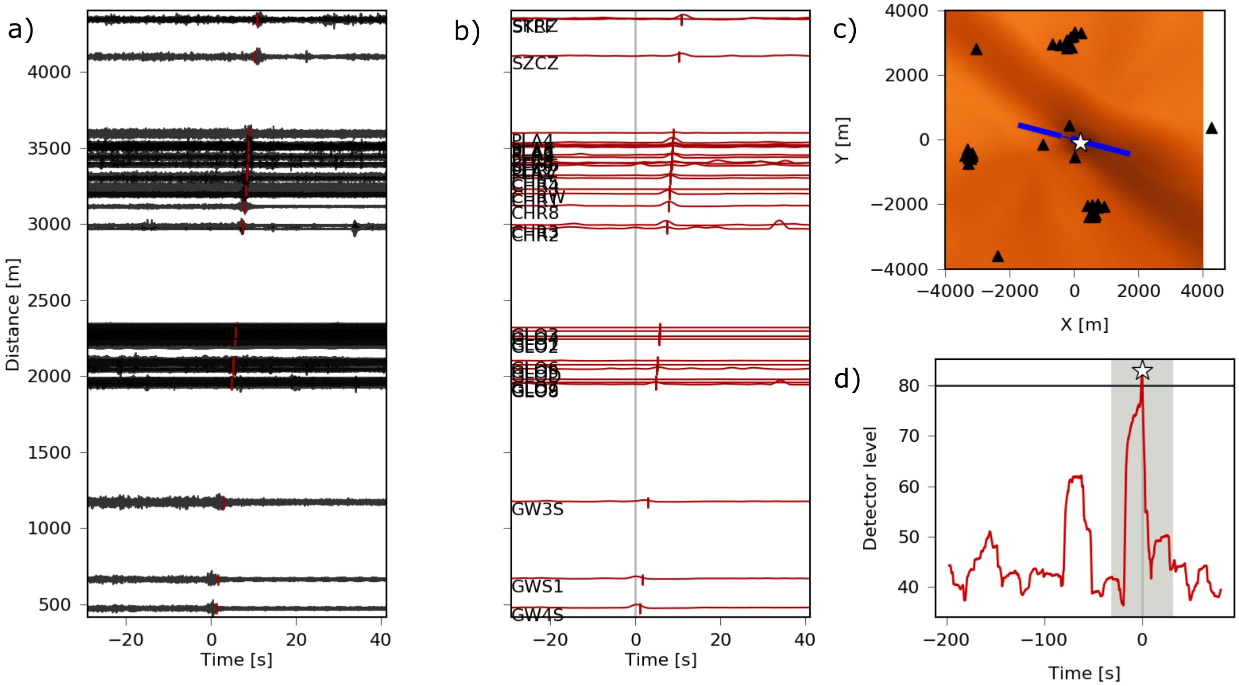

Figure S10. Hypocentral location on 2016 August 31th, 23:08:47 UTC time (Method M5). a) Waveforms sorted by hypocentral distance. b) Characteristic function (normalized amplitude envelopes) for each trace. These are used for travel–time stacking corrected with S-wave speed (red lines). The markers indicate the (best fit) synthetic arrival time of the S-phase at each sensor. c) Coherence (stack) map for the search region. Dark colors denote high coherence values. A white star marks the location of the detected event. Sensor locations are shown with black triangles. d) Global detector level function in a processing time window from -20 to 20 s around the origin time of the detected event. The cutout time window used for the coherence map is shown in gray color. White star indicates the detection exceeding a detector level threshold of 80.

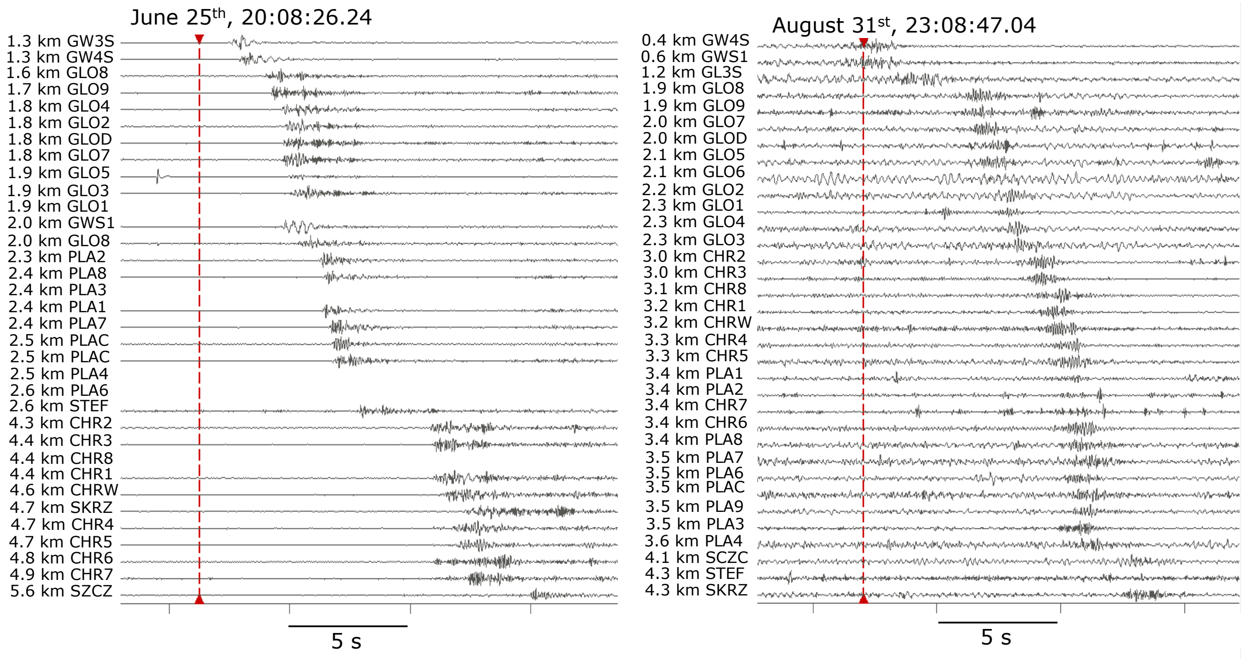

Figure S11. Waveforms sorted by hypocentral distance for the detected events on 2016 June 25th (left) and August 31th (right). Source-receiver distance in km and the seismic station name are indicated in the label of each trace. Waveforms are band-pass filtered in the frequency range 2–15 Hz.

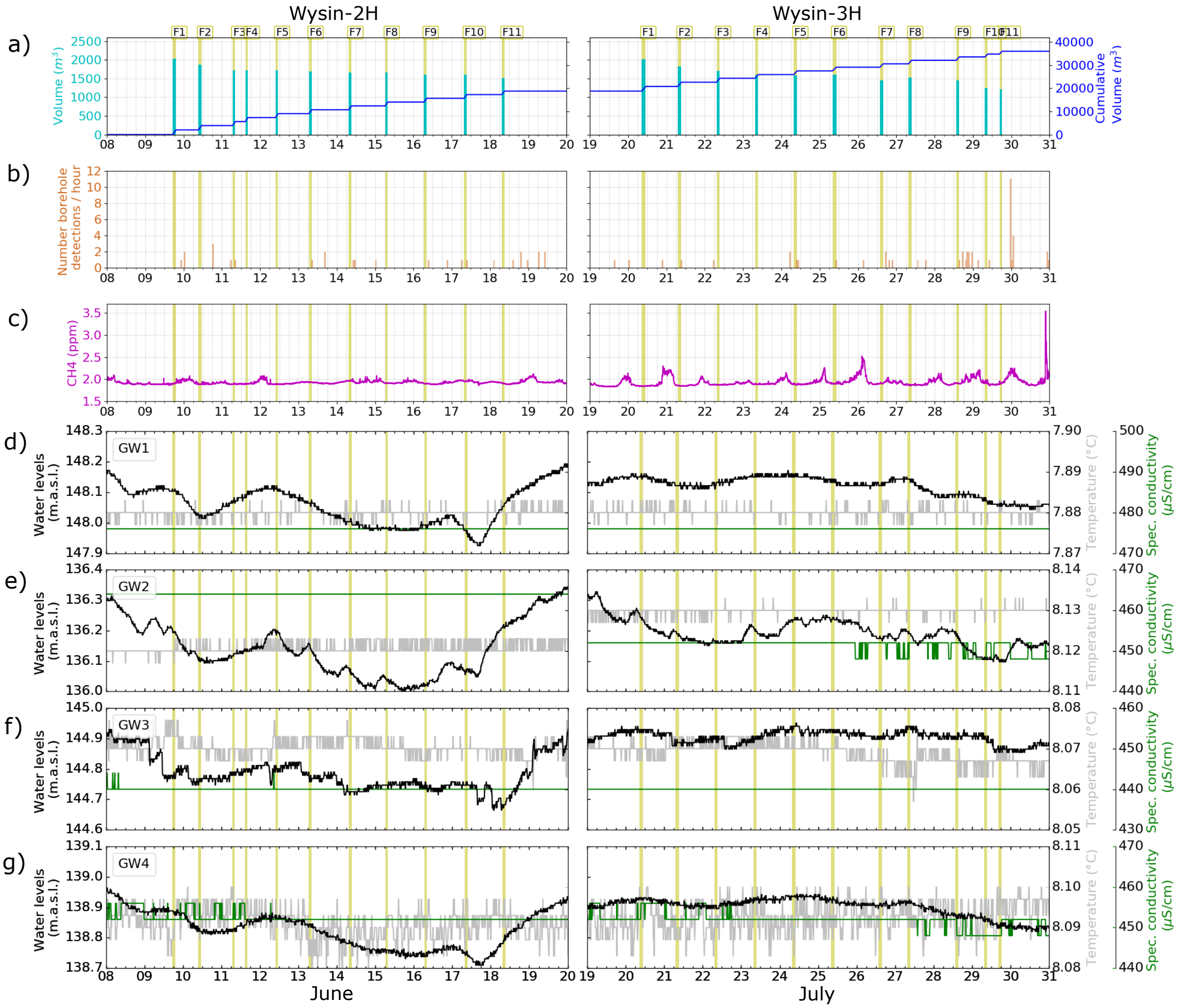

Figure S12. Zoom in Figure 8 around the HF stimulations at Wysin-2H (left) and Wysin-3H (right). Correlation among fluid volumes injected, seismicity, air pollution and groundwater conditions. Yellow bands indicate the SNAI duration associated to each frac stage (F1 to F11). Time marks are at 6-hour intervals. (a) Fluid volumes injected in each frac stage and the cumulative volume. (b) Distribution of local HF detections per hour. (c) Methane content (CH_4) in ppm. (d-h) Water levels, temperature and specific conductivity (black, gray and green line, respectively) for each groundwater borehole. Note the same height is shown in y-axes for the water levels (0.4 m), temperature (0.03°C) and specific conductivity (30 μS/cm).

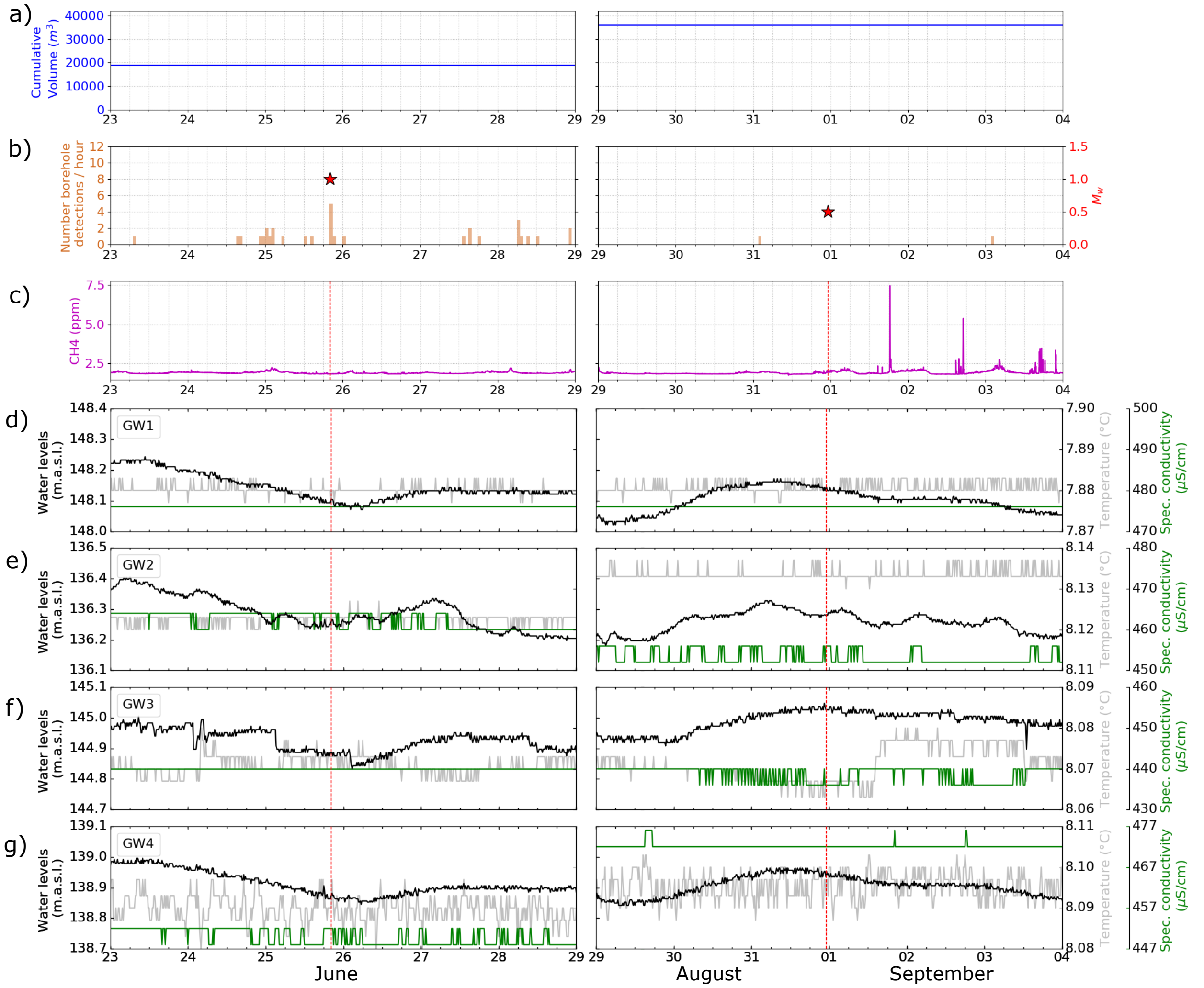

Figure S13. Zoom in Figure 8 during 6-day intervals around the largest seismic events recorded after HF operations at the Wysin site: the M_w 1.0, 2016 June 25th (left) and the M_w 0.5, 2016 August 31st (right). Correlation among fluid volumes injected, seismicity, air pollution and groundwater. Time marks are at 6-hour intervals. (a) Cumulative volume injected. (b) Distribution of local HF detections per day (left axis) and the located events with M_w (red stars, right axis). (c) Methane (CH_4) in ppm. (d-h) Water levels, temperature and specific conductivity (black, gray and green line, respectively) for each groundwater borehole. Note the same height is shown in y-axes for the water levels (0.4 m), temperature (0.03°C) and specific conductivity (30 μS/cm). Vertical red dashed lines in c) to g) indicate the time of the largest seismic events according the red stars in b).

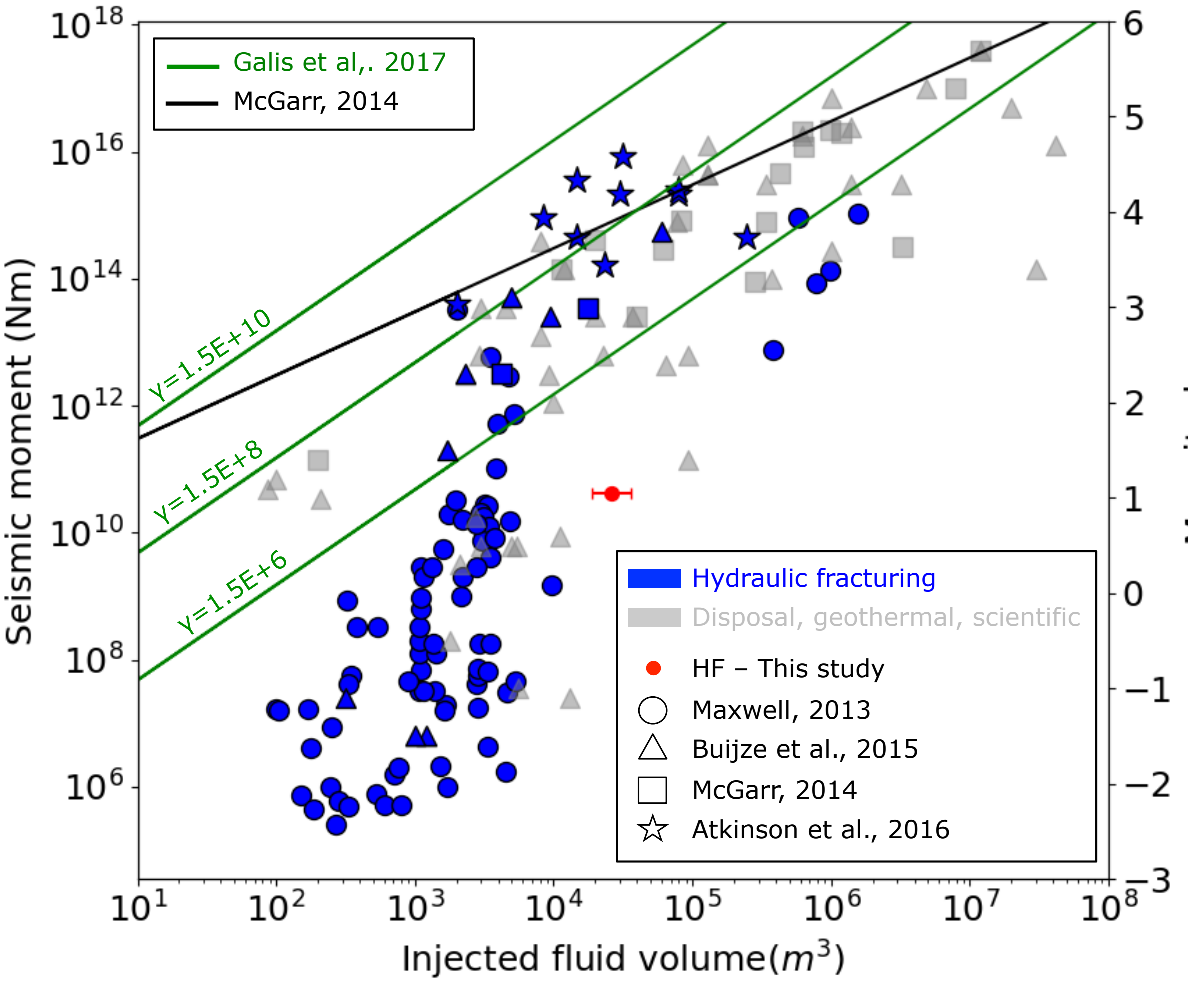

Figure S14. Total volume of injected fluid from the start of injection until the time of the largest induced earthquake versus seismic moment (in N·m on left axis, equivalent moment magnitude on the right axis). Observations of different induced seismicity cases[6,7,8,9] including hydraulic fracturing (blue color) and others (wastewater disposal, geothermal and scientific projects - gray color). For completeness, we also include the maximum magnitude predicted by McGarr[8] [$M_0^{max} = G\Delta V$, (G = 3e10 Pa)] and Galis et al.[10] [$M_0^{max-arr} = \gamma \Delta V^{3/2}$]. Hydraulic fracturing result in this study is indicated by a red dot, with error bars that show the range of injected volume from the stage prior to event occurrence (minimum) to the sum of volumes for all stages (maximum).

Depth (m)		Chronostratigraphy		Main formations
from	to	Period	Stage	
0	158	Quaternary		quartz sand, gravels, silts, clays
158	225	Neogen & Paleogen		quartz sand, silts, geza, limestone, granite, boulder clay
225	293	Cretaceous	Maastrichtian	quartz sand, gravel, marls
293	501,5		Campanian	calcareous sandstone, marl, geza
501,5	607		Santonian	quartz-glauconitic sandstones
607	640		Coniacian	quartz-glauconitic sandstones, geza, mudstones
640	792		Turonian	gray mudstone
792	810		Cenomanian	siltstone, quartz sandstone
810	838,5		Albian	sand, sandstone, gravel, siltstone
838,5	875	Jurassic	Kimmeridgian	siltstone
875	1027,5		Oxfordian	oolitic limestone, sandy limestone, sandy siltstone, clay mudstones
1027,5	1035		Callovian	clays, calcareous sandstones
1035	1047,5		Bajocian	siltstones, sandstones
1047,5	1051		Dogger	siltstones, sandstones
1051	1089		Pliensbachian	sandstone
1089	1108	upper Triassic	Rhaetian	siltstones, claystones, sandstones
1108	1177,7	middle Triassic	Muschelkalk top, middle, bottom	siltstones, dolomite, claystone, sandstone, marl
1177,7	1699	lower Triassic	Buntsandstein	limestone, sandstone, siltstone, claystone
1699	2095,3	Upper Permian	Main annhydrite A3	mudstones, anhydrite, dolomite, rock salt, limestone
2095,3	2173	Silurian	Lower Podlasie	claystone
2173	2560		Upper Siedlce	claystone
2560	2745		Middle Siedlce	claystone, siltstone
2745	3360		Lower Siedlce	claystone, siltstone
3360	3960		Upper Mielnik	claystone, siltstone
3960	4205		Lower miernik	claystone, siltstone
4205	4302,5		Upper Bielsk	claystone
4302,5	4326,5		Lower Bielsk	claystone
4326,5	4398		Llandovery	claystone, black tarry and bitumen shale, sandstone, limestone
4398	4403	Ordovician	Katian – Aszgil	claystone
4403	4418		Caradok	claystone, bentonite, limestone
4418	4419,5		Landeil	limestone
4419,5	4421,1		Landwirn	limstone, claystone
4421,1	4438,9		Arenig	claystone, sandstone, siltstone
4438,9	4748	Cambrian	Center Cambrian	siltstone, sandstone, claystone
4748	5026,7		Lower Cambrian	sandstone, siltstone, claystone
5026,7	5154,9	Proterozoic	Ediacaran	sandstone, enderbit
5154,9	5202		Precambrian	enderbit, mylonite

Table S1. Lithology-stratigraphic profile for the Koscierzyna IG-1 research borehole located to 8.25 km from the Wysin's wellhead[11].

References

1. P. I. Geologiczny & P. I. Badawczy Polish Geological Institute – National Research Institute]. Assessment of shale gas and shale oil resources of the lower Paleozoic Baltic-Podlasie-Lublin Basin in Poland. First report. Warsaw, Poland. 29 pp (2012), https://www.pgi.gov.pl/en/dokumenty-pig-pib-all/aktualnosci-2012/zasoby-gazu/769-raport-en/file.html (last accessed 28 March 2018).
2. Pokorski, J. Geological section through the lower Paleozoic strata of the Polish part of the Baltic region. Geological Quarterly 54(2),123–130 (2010).
3. Państwowy Instytut Geologiczny – Państwowy Instytut Badawczy, Akademia G.rniczo-Hutnicza w. Krakowie & Politechnika Gdańska [Polish Geological Institute – National Research Institute, AGH University of Science and Technology in Krak.w & Gdańsk University of Technology]. Określenie zakresu oddziaływania procesu poszukiwania i eksploatacji niekonwencjonalnych zł.ż węglowodor.w na środowisko, z uwzględnieniem termin.w poszczeg.lnych prac, infrastruktury podziemnej i przesyłowej, a w szczeg.lności określenia charakteru i zakresu oddziaływania: RAPORT KOŃCOWY z realizacji badań na poligonie badawczym nr 4 – Wysin [Assessment of effects of exploration and recognition of the unconventional hydrocarbon deposits considering the execution schedule, underground and transport infrastructure, specifically the character and scope of such effects. Final report on tests executed at the Wysin test ground No. 4]. Warsaw, Poland. 111 pp. (in Polish), (2014).
4. Grad, M., Polkowski, M. & Ostaficzuk, S. R. High-resolution 3D seismic model of the crustal and uppermost mantle structure in Poland. Tectonophysics 666, 188–210 (2015).
5. Grabowska, T., Bojdys, G. & Dolnicki, J. Three-dimensional density model of the Earth's crust and the upper mantle for the area of Poland. J. Geodyn. 25, 5–24 (1998).
6. Maxwell, S. C. Unintentional seismicity induced by hydraulic fracturing, CSEG Rec. - Focus Artic. 38(8) (2013).
7. Buijze, L., Wassing, B., Fokker, P. A. & va Wees, J. D. Moment partitioning for injection induced seismicity: Case studies and insights from numerical modeling, in Proceedings World Geothermal Congress 2015, Melbourne, Australia, 19 to 25 April 2015.
8. McGarr, A. Maximum magnitude earthquakes induced by fluid injection. J. Geophys. Res. 119, 1008–1019 (2014).
9. Atkinson, G. M. et al. Hydraulic Fracturing and Seismicity in the Western Canada Sedimentary Basin. Seismol. Res. Lett. 87, 631–647 (2016).
10. Galis, M., Ampuero, J. P., Mai, P. M. & Cappa, F. Induced seismicity provides insight into why earthquake ruptures stop. Science Advances 3, eaap7528 (2017).
11. Marcak, H., Mirek, J. & Lasak, M. Use the seismic interferometry method to assess the effects of hydrofracturing in Wysin boreholes. Seventh EAGE Workshop on Passive Seismic 2018, Krakow, Poland, 26 to 29 March 2018.
